# 100‐year time series reveal little morphological change following impoundment and predator invasion in two Neotropical characids

**DOI:** 10.1111/eva.12763

**Published:** 2019-02-27

**Authors:** Ilke Geladi, Luis Fernando De León, Mark E. Torchin, Andrew P. Hendry, Rigoberto González, Diana M.T. Sharpe

**Affiliations:** ^1^ Redpath Museum and Department of Biology McGill University Montreal Quebec Canada; ^2^ Department of Biology University of Massachusetts Boston Boston Massachusetts; ^3^ Centro de Biodiversidad y Descubrimiento de Drogas Instituto de Investigaciones Científicas y Servicios de Alta Tecnología (INDICASAT‐AIP) Panama Republic of Panama; ^4^ Smithsonian Tropical Research Institute Balboa, Ancon, Panama Republic of Panama

**Keywords:** geometric morphometrics, impoundment, invasive species, Lake Gatun, maladaptation, morphological change, multiple stressors

## Abstract

Human activities are dramatically altering ecosystems worldwide, often resulting in shifts in selection regimes. In response, natural populations sometimes undergo rapid phenotypic changes, which, if adaptive, can increase their probability of persistence. However, in many instances, populations fail to undergo any phenotypic change, which might indicate a variety of possibilities, including maladaptation. In freshwater ecosystems, the impoundment of rivers and the introduction of exotic species are among the leading threats to native fishes. We examined how the construction of the Panama Canal, which formed Lake Gatun, and the subsequent invasion of the predatory *Cichla monoculus* influenced the morphology of two native fishes: *Astyanax ruberrimus* and *Roeboides* spp. Using a 100‐year time series, we studied variation in overall body shape over time (before vs. after impoundment and invasion) as well as across space (between an invaded and an uninvaded reservoir). In addition, we examined variation in linear morphological traits associated with swim performance and predator detection/avoidance. Notwithstanding a few significant changes in particular traits in particular comparisons, we found only limited evidence for morphological change associated with these two stressors. Most observed changes were subtle, and tended to be site‐ and species‐specific. The lack of a strong morphological response to these stressors, coupled with dramatic population declines in both species, suggests they may be maladapted to the anthropogenically perturbed environment of Lake Gatun, but direct measures of fitness would be needed to test this. In general, our results suggest that morphological responses to anthropogenic disturbances can be very limited and, when they do occur, are often complex and context‐dependent.

## 1 INTRODUCTION

Humans are altering ecosystems across the globe. Species introductions, climate change, and habitat modification (Ditchkoff, Saalfeld, & Gibson, 2006; Palumbi, 2001; Sala, Piper, & Hoch, 2010; Stockwell, Hendry, & Kinnison, 2003) are leading causes of biodiversity loss worldwide (Wood, Stedman‐Edwards, & Mang, 2000). These activities also likely impose strong, novel selective pressures on natural populations (but see Fugère & Hendry, 2018). In response, populations sometimes undergo rapid phenotypic changes (Hendry, Farrugia, & Kinnison, 2008; Sharpe & Hendry, 2009; Strauss, Lau, & Carroll, 2006), which, if adaptive, can increase their probability of persistence (i.e., evolutionary rescue, Bell & Gonzalez, 2011; Derry et al., 2019; Gomulkiewicz & Holt, 1995).

However, in many instances, populations might fail to undergo phenotypic change following an anthropogenic perturbation. A lack of phenotypic change might be maladaptive, ultimately resulting in population declines, or even extinctions (Balirwa et al., 2003; Strauss et al., 2006). The inability to adapt to a novel selective pressure could occur for a multitude of reasons (Crespi, 1999; Hendry & Gonzalez, 2008). For example, the focal population might possess insufficient genetic variation in the traits under selection, or maladaptive alleles might be introduced through mutation, drift, or gene flow (Hendry, Taylor, & Mcphail, 2002; Lewontin, 1974). Alternatively, the environmental change might be too abrupt, or too extreme (e.g., Rolshausen et al., 2015), and/or might impose conflicting or varying selective pressures (e.g., Sharpe & Chapman, 2018). Adaptation could be further hindered by multiple or indirect species interactions (Benard, 2006), and fluctuating population demographics (Lau & Terhorst, 2015). However, a lack of morphological change might not always imply maladaptation. For example, species might not change following a perturbation because they are already pre‐adapted in some way. They might also successfully avoid or buffer the effects of the stressor through other means, such as migration, or habitat or niche shifts (Archard, Earley, Hanninen, & Braithwaite, 2012; Werner, Gilliam, Hall, & Mittelbach, 1983; Zaret & Suffern, 1976), thus weakening or eliminating selection on the phenotype. It is important to have a better understanding of the ability of species to adapt (or not) in response to human disturbances to better predict how they will persist in the face of increasing anthropogenic change (Hendry et al., 2008).

In freshwater habitats, two of the greatest threats to native biodiversity are habitat modification through diversion and impoundment of natural watercourses, and introduced species, which often occur hand in hand (Franssen, Harris, Clark, Schaefer, & Stewart, 2012; Hall & Mills, 2000; Turgeon, Turpin, & Gregory‐Eaves, 2018; Vörösmarty et al., 2010). Impoundment through dams currently affects an estimated two‐thirds of freshwater rivers globally (Nilsson & Berggren, 2000) and can impose selection on freshwater organisms in a number of ways, including through restricting migration and altering water flow, temperature regimes, and sediment transport (Fukushima, Kameyama, Kaneko, Nakao, & Steel, 2007; Liermann, Nilsson, Robertson, & Ng, 2012; Nilsson & Berggren 2000). Impoundments can also facilitate the invasion of exotic species (Johnson, Olden, & Vander Zanden, 2008) such as top predators, which tend to have the strongest ecological impacts (DiDonato & Lodge, 1993; Vega‐Trejo, Zuniga‐Vega, & Langerhans, 2014) on freshwater ecosystems (Balirwa et al., 2003; Chapman et al., 1996; Findlay, Bert, & Zheng, 2000; Sowersby, Thompson, & Wong, 2015; Zaret & Paine, 1973). For instance, predator introductions can lead to declines or local extinctions of native species (Balirwa et al., 2003; Findlay et al., 2000), changes in fish habitat use and behavior (Chapman et al., 1996; Sowersby et al., 2015), and alteration of food availability and nutrient dynamics (Sowersby et al., 2015; Vitule, Freire, & Simberloff, 2009). While the ecological consequences of these stressors are well understood, we still know relatively little regarding the extent to which they may influence trait evolution in species that do manage to persist.

Here, we examine whether evidence exists of morphological change in two Neotropical fishes (*Astyanax ruberrimus* and *Roeboides* spp.) (Eigenmann, 1913; Gunther, 1864; Meek & Hildebrand, 1916) following the impoundment of the Chagres River to form Lake Gatun in Panama in 1914, and the 1967 introduction of a novel piscivore, *Cichla monoculus* (peacock bass) (Agassiz, 1831). We focused on external morphology and body shape because these aspects have been well studied in fishes (Langerhans & Reznick, 2010; Walker, 1997; Webb, 1977) and often show predictable, parallel evolutionary responses to divergent hydrological and predation regimes (Table 1) (Chivers, Zhao, Brown, Marchant, & Ferrari, 2008; Klepaker, 1993; Kristjánsson, 2005; Langerhans, 2008; Langerhans & Reznick, 2010; Ravinet, Prodöhl, & Harrod, 2013). We envisioned three potential scenarios. First, both native species might show substantial, parallel morphological changes following impoundment and predator introduction that matched a priori expectations, which would suggest a potentially adaptive (plastic or genetic) response to these stressors. Second, both species might show no change at all, suggesting that for any of the reasons listed above, they were unable to adapt, or that their existing morphology was pre‐adapted to cope with these stressors. Third, both species could show subtle and/or contrasting morphological changes, suggesting that local environmental factors may be interacting with impoundment and invasion to shape the morphological response.

**Table 1:** Expected and observed morphological trends in response to impoundment and an increase in predation (with invasion). Observed trends are reported for an effect size greater than 10%. If effect size was less than 10%, it is reported as “no substantial change.” The direction of trends refers to the expected shift in the perturbed (impounded/invaded) population relative to the unperturbed population. Cases where our results matched expected trends are highlighted in bold

	Overall body form	Body depth (BD)	Anterior body depth (AD)	Caudal peduncle (CPA, CPD)	Eye area (EA)	Caudal spot area (CSA)
*Impoundment*						
Predicted trend	Taxon‐specific^a^	Taxon‐specific^a^	Shallower^b^	Taxon‐specific^c^	Unclear^d^	Unclear^d^
Observed *Astyanax*	No change	No substantial change	No substantial change	No substantial change	Decrease (Mandinga)	Increase (Mandinga, Trinidad, Chagres)
Observed *Roeboides*	Deeper‐bodied	Deeper	**Shallower**	Deeper	Increase (Chagres)	Increase (Frijoles)
*Predation*						
Predicted trend	Smaller anterior region; larger/deeper mid‐body and caudal region^e^	Deeper^f^	Shallower^g^	Increase^h^	Unclear^i^	Increase^j^
Observed *Astyanax*	Mixed results: **Shallower head and body but larger caudal peduncle regions in Chagres over time,** but no change in Gatun over time; no difference between Gatun and Bayano	Shallower mid‐body (Chagres over time)	**Shallower heads (Chagres over time)**	Increase (Chagres over time)	No change	Decrease (Gatun over time)
Observed *Roeboides*	Mixed results: **Deeper bodies in Gatun**, shallower bodies in Chagres; Gatun shallower bodies than Bayano	**Deeper (Gatun over time);** Shallower (Chagres over time; Gatun across space)	**Shallower (Gatun over time and across space);** Longer (Chagres over time)	**Increase (Gatun over time and across space);** Decrease (Chagres over time)	Increase (Chagres over time)	**Increase (Gatun over time)**

a Body shape variation among lotic (flowing) and lentic (still) waters has been found to be taxon‐specific. Body depth was greater in lentic environments in cyprinids (Franssen, 2011; Haas et al., 2010), cichlids, and characids (Langerhans, 2008; Langerhans et al., 2003), but smaller in Gasterosteidae (Hendry et al., 2002; Sharpe et al., 2008) and Salmonidae (Pakkasmaa & Piironen, 2001).

b Fish in lentic environments typically have smaller/shallower heads compared to fish in lotic/riverine environments (Franssen, 2011; Franssen et al., 2012; Haas et al., 2010; Langerhans et al., 2003; Pakkasmaa & Piironen, 2001).

c Caudal peduncles became deeper in Cichlidae/Characidae (Langerhans et al., 2003), and shallower but longer in 3 other families (Krabbenhoft et al., 2009) following creation of a lake.

d Reservoirs/lakes present a very different visual environment than more turbid streams and rivers, which could alter the costs and benefits associated with visual signaling (caudal spots) and organs (eyes), but the direction of change is difficult to predict a priori.

e Fish in high‐predation environments generally have deeper, less streamlined bodies, to improve unsteady swimming behavior, including fast‐starts (Langerhans & Reznick, 2010).

f Deeper bodies may help deter gape‐limited predators (Domenici et al., 2008; Lönnstedt et al., 2013), misdirect strikes (Webb, 1986), or increase performance in escape maneuvers (fast‐starts) (Langerhans & Reznick, 2010; Law & Blake, 1996).

g Fish in high‐predation environments typically have shallower heads (Langerhans & DeWitt, 2004; Langerhans & Reznick, 2010).

h Deeper/longer/larger caudal peduncles are associated with increased thrust and fast‐start escape performance, which is important during predator escape (Langerhans & DeWitt, 2004; Langerhans et al., 2004; Langerhans & Reznick, 2010).

i Eyes might be expected to get larger (to improve predator detection) or smaller (to improve crypsis) (Lönnstedt et al., 2013).

j Caudal spots have been proposed as antipredator defenses because they draw predator strikes away from the head (Kjernsmo & Merilaita, 2013; Lönnstedt et al., 2013), reduce cannibalism (Zaret, 1977), and reduce fin predation (Winemiller, 1990).

This study system provides an excellent opportunity to study contemporary phenotypic responses to multiple stressors. Lake Gatun has a long history of ichthyological collections, dating back to surveys conducted prior to the construction of the Panama Canal in the early 20th century. These collections provide an opportunity to evaluate morphological change in native species over a roughly 100‐year period. Furthermore, the history of Lake Gatun is representative of many Neotropical drainages, where rivers have first been impounded and then stocked with (or invaded by) exotic species. The availability of historical specimens from before and after both stressors allows us to develop a comprehensive set of spatial and temporal comparisons to disentangle these two stressors in a way that is not typically possible.

## 2 MATERIALS AND METHODS

### 2.1 Study design

To test for morphological change within each taxon (*Astyanax ruberrimus*,* Roeboides* spp.), we carried out four types of comparisons (Table 2). First, to assess the potential impact of impoundment, we compared historical stream specimens to specimens from Lake Gatun soon after its formation but *before* the introduction of peacock bass (*a: impoundment effect*). Second, we compared preversus postintroduction specimens from two invaded populations (*b: invasion effect through time*). Third, we compared contemporary specimens between invaded (Gatun) versus uninvaded (Bayano) reservoirs (*c: invasion effect across space*). Finally, to assess variation in morphology over time in the absence of human interventions, we compared historical versus contemporary specimens from two independent, uninvaded streams within the same watershed (*d: temporal control*).

**Table 2:** Study design: comparisons used to test our questions of interest

Question	Specific comparisons
(a) Impoundment effect: Compare tributary streams of Gatun versus Gatun postimpoundment (but pre‐*Cichla*)	
*Astyanax ruberrimus*	Tributary streams (Mandinga Stream 1911, Trinidad Stream 1911, Chagres River 1911) versus newly formed reservoir (Gatun 1935)
*Roeboides guatemalensis*	Tributary streams (Mandinga Stream 1911, Frijoles Stream 1911, Chagres River 1911) versus newly formed reservoir (Gatun 1935)
(b) Invasion effect through time: Compare populations pre‐ versus post‐*Cichla* introduction	
*Astyanax ruberrimus*	Lake Gatun (1935) versus Lake Gatun (2013)
	Chagres River (1911) versus Chagres River (2013)
*Roeboides guatemalensis*	Lake Gatun (1935) versus Lake Gatun (2013)
	Chagres River (1911) versus Chagres River (2002)
(c) Invasion effect across space: Compare contemporary invaded (Gatun) versus uninvaded (Bayano) reservoirs	
*Astyanax ruberrimus*	Lake Gatun (2013) versus Lake Bayano (2013)
*Roeboides* spp.	Lake Gatun (2013) versus Lake Bayano (2013)
(d) Temporal controls: Compare populations in tributary streams that have experienced neither impoundment nor invasions over time	
*Astyanax ruberrimus*	Trinidad Stream (1911) versus Trinidad Stream (2014)
	Mandinga Stream (1911) versus Mandinga Stream (1994)
*Roeboides guatemalensis*	Mandinga Stream (1911) versus Mandinga Stream (1992)
	Frijoles Stream (1911) versus Frijoles Stream (1998)

### 2.2 Study sites

**Figure 1:**
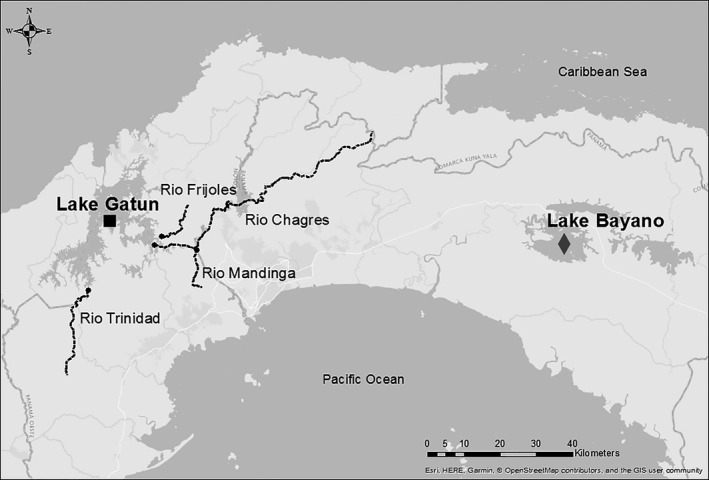
Map of study sites. Fish were sampled from Lake Gatun (black square; impounded + invaded), Lake Bayano (gray diamond; only impounded), and four rivers in the Chagres watershed (black circles/dotted black lines)

All of the above comparisons were carried out using freshwater populations from the Chagres and Bayano watersheds in Panama (Figure 1). The Chagres River was damned in 1910 to create Lake Gatun and the Panama Canal, which was completed in 1914 (Keller & Stallard, 1994). Both were quickly colonized by native riverine fishes (Smith, Bell, & Bermingham, 2004). Peacock bass were introduced to Panama for sport fishing in 1965, and subsequently escaped and invaded the Chagres River, reaching Lake Gatun in 1967 (Zaret & Paine, 1973). Zaret and Paine (1973) showed that almost immediately following the introduction, peacock bass eliminated six of the eight previously common native fish species and drastically reduced the abundance of the seventh (Zaret & Paine, 1973). Recent work has shown that the fish community has failed to recover in the intervening 45 years and that the abundance of almost all small‐bodied native fishes, including *A. ruberrimus* and *Roeboides* spp., remains at extremely low levels (Sharpe, De León, González, & Torchin, 2017).

Other exotic piscivores have invaded Lake Gatun in the last ten years (*Astronotus ocellatus*,* Parachromis managuensis*); however, peacock bass remains the dominant predator, both in terms of abundance and biomass (Sharpe et al., 2017). Native predators in the Chagres watershed include *Hoplias* spp., *Gobiomorus* spp., *Eleotris* spp., *Rhamdia* spp., and *Synbranchus marmoratus*. Of these, three are nocturnal, three are omnivorous, and all are benthic, ambush predators (Bussing, 2002; Zaret & Rand, 1971). Given the strategies of native predators, we expected that the introduction of a highly piscivorous diurnal pursuit predator like the peacock bass (Sharpe et al., 2017) would represent a stronger, and novel, selection pressure for native prey.

The two invaded populations (Chagres River, Lake Gatun) were compared to three smaller tributaries of Gatun, which peacock bass failed to colonize, and to Lake Bayano, another large reservoir in Eastern Panama, which has remained uninvaded (Table 3). The main predators in Lake Bayano are the native *Hoplias malabaricus* and *Ctenolucius beani* (Table 3). Despite small‐scale variation within sites, environmental attributes do not vary greatly across watersheds (Sharpe et al., 2017), and sites are located in the same climate zone, that being lowland tropical forest in central Panama (Angermeier & Karr, 1983).

**Table 3:** Summary of characteristics and species in lakes and rivers used in study. “N/A” means information not available. Data were collected from De León, *pers. comm*.; Gonzalez, *pers. comm*.; and Angermeier and Karr (1983), Gutiérrez et al. (1995), Hildebrand (1938), Homans (1835), Mattox, Bifi, and Oyakawa (2014), Sharpe et al. (2017), and Smith et al. (2004)

Attribute	Gatun	Bayano	Chagres	Frijoles	Mandinga	Trinidad
Habitat Type	Lake	Lake	River	Stream	Stream	Stream
River Drainage	Chagres	Bayano	Chagres	Chagres	Chagres	Chagres
Introduced Piscivores	*Cichla monoculus, Parachromis managuensis*	None	*Cichla monoculus, Parachromis managuensis*	None	None	None
Other introduced species	*Astronotus ocellatus, Mesonauta festivus, Oreochromis niloticus, Tilapia rendalli, Gambusia holbrooki*	*Oreochromis niloticus*	*Astronotus ocellatus, Mesonauta festivus, Oreochromis niloticus*	*N*/A	*N*/A	*N*/A
Mean depth (m)	13	13.6	8.1	0.2	*N*/A	0.2
Surface Area (km^2^)	407.4	350	*N*/A	*N*/A	*N*/A	*N*/A
Year Created	1910–1914	1976	Natural waterbody	Natural waterbody	Natural waterbody	Natural waterbody

### 2.3 Study populations

*Astyanax* and *Roeboides* are highly diverse genera of small‐bodied characid fishes that are widespread across Central America (Bussing, 2002). *Astyanax* is a genus of surface‐dwelling fish that feeds mainly on terrestrial and aquatic invertebrates as well as terrestrial plant matter (Angermeier & Karr, 1983; Zaret & Rand, 1971). *Roeboides* are specialized scale‐eaters, although they also feed on aquatic invertebrates (Angermeier & Karr, 1983; Peterson & Winemiller, 1997). Five species of *Astyanax* are found in Panama, with *Astyanax ruberrimus* (our focal species) being very widespread, and found on both sides of the continental divide (Smith & Bermingham, 2005). For *Roeboides*,* Roeboides occidentalis* (the Pacific sister species) is found exclusively in the Bayano drainage, whereas *Roeboides guatemalensis* (the Atlantic species) is found in the Chagres drainage (Lake Gatun, Chagres River and their tributaries). Thus, for *Roeboides*, we conducted spatial comparisons at the level of the genus. However, previous morphological analyses by Meek and Hildebrand (1916), including many of the same linear traits we measure here, indicate that these two species only differ in two meristic traits (number of lateral lines scales and gill rakers), coloration (a round blotch vs. a longitudinal bar), and slight changes in the origin of dorsal and anal fins, but not in overall shape or size.

### 2.4 Fish collections

Contemporary specimens of *A. ruberrimus* and *Roeboides* spp. were collected between 2013 and 2015 (Supporting Information Table S1). Fish were captured using various methods, including minnow traps, cast‐nets, and multipanel experimental gillnets (monofilament, 45.7 m long, 3 m deep, 6 panels with stretched mesh ranging from 2.54 cm to 15.24 cm). After capture, fish were immediately euthanized with clove oil, following animal care protocols approved by the Smithsonian Tropical Research Institute (Protocol # 2013‐0507‐2016). Specimens were then fixed in 10% formalin for at least a week before being preserved in 70% ethanol for morphological analyses. The only exception was for *A. ruberrimus* from the Trinidad Stream, for which fixed contemporary specimens were not available, and thus, photographs of fresh specimens were used. Shrinkage due to preservation is minimal for gross morphology (e.g., fish standard length is known to shrink proportionally with body depth to small degrees (0.8%–4%); Gaston, Jacquemin, & Lauer, 2013; Kristoffersen & Salvanes, 1998); therefore, we do not think that the use of fresh specimens for this single population influenced our results in any substantial manner. Whenever possible, we used an even representation of specimens from each year for analyses, aiming for a total of approximately 30 individuals per population (Supporting Information Table S1). In a few cases, samples from multiple years were pooled to increase sample sizes (Supporting Information Table S1). In those instances, we first plotted data separately by year (not shown), but means were very similar; therefore, data were pooled for subsequent analyses.

Historical specimens of *A. ruberrimus* and *R. guatemalensis* were photographed, with permission, from collections at the Smithsonian National Museum of Natural History (NMNH) in Washington, DC, and the Neotropical Fish Collection at the Smithsonian Tropical Research Institute (STRI) in Panama (Supporting Information Table S1). All historical specimens had been fixed in 10% formalin and then stored in 70% ethanol. Studies have shown that long‐term preservation has minimal effects on most aspects of fish morphology (Gaston et al., 2013; Kristoffersen & Salvanes, 1998); thus, we do not think that length of fish preservation introduced any substantial bias. We were not able to dissect museum specimens to directly determine their sex and maturity status; therefore, these factors were not explicitly addressed in our analyses. However, because fish specimens used in the study were selected at random, we do not expect that these factors caused a systemic bias in our results.

### 2.5 Morphological analyses

#### 2.5.1 Overall body shape

**Figure 2:**
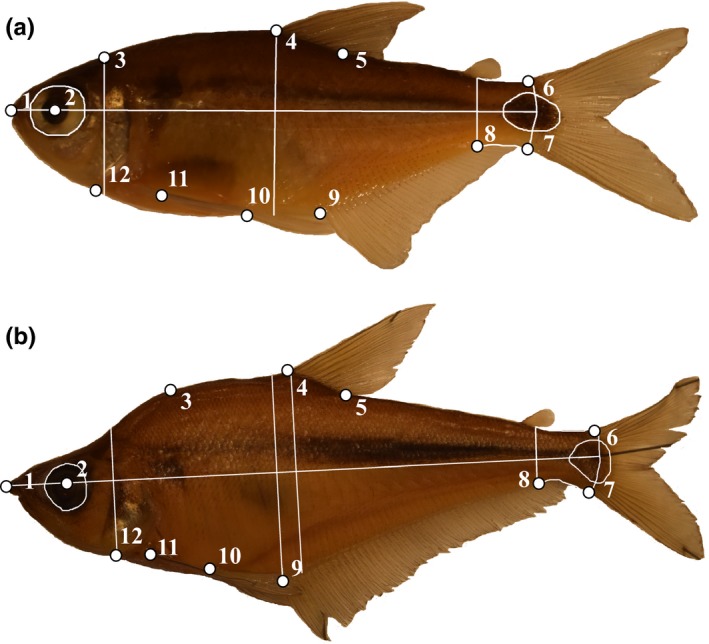
Measurements taken in ImageJ for the individual trait analysis and the twelve homologous landmarks used for geometric morphometrics (see text for details). Panel A shows *A. ruberrimus*. Panel B shows *R. guatemalensis*

Preserved fish were laid flat on a grid and photographed using a digital camera mounted on a tripod. When necessary, small pins were used to extend the median and caudal fins. Variation in overall body shape was examined using geometric morphometrics, a tool that uses Cartesian coordinates to describe, visualize, and quantify shape variation (Adams & Otárola‐Castillo, 2013; Zelditch, Swiderski, & Sheets, 2012). We digitized the following 12 homologous landmarks on the lateral body profile of images (Figure 2) using TPSDig2 (Rohlf, 1999): (1) most anterior point of the premaxilla, (2) center of the eye orbit, (3a) in *A. ruberrimus*, the top insertion of the most anterior gill cover, (3b) in *Roeboides* spp., the small indentation at the mark where the hump peaked, (4) anterior insertion point of the dorsal fin, (5) posterior insertion of the dorsal fin, (6) dorsal insertion point of the caudal fin, (7) ventral insertion point of the caudal fin, (8) posterior‐most point where the anal fin meets the body, (9) anterior insertion point of the anal fin, (10) insertion point of the pelvic fin, (11) dorsal insertion point of the pectoral fin, and (12) intersection of the operculum and body profile (Sharpe, Langerhans, Low‐Decarie, & Chapman, 2015).

Photographs were all landmarked by the same individual (I. Geladi). Landmark configurations were translated to the origin, scaled to unit‐centroid size, and rotated using generalized Procrustes analysis in the Geomorph package in R (Adams & Otárola‐Castillo, 2013; Sherratt, 2014). These landmarks were then projected into a linear tangent space, yielding Kendall's tangent space coordinates (Dryden & Mardia, 1993; Rohlf 1999). Next, we used the plotTangentSpace function in Geomorph to plot these specimens along their principal axes and perform a principal components analysis (PCA). Finally, we visualized shape differences between groups using the plotRefToTarget function.

#### 2.5.2 Linear traits

We used ImageJ (Rasband, 1997) to measure the following seven linear traits (Figure 2): (a) standard length (SL), (b) eye area (EA), (c) body depth at anterior insertion point of dorsal fin (BD), (d) anterior depth (AD), (e) caudal peduncle depth (CPD), (f) caudal peduncle area (CPA), and (g) caudal spot area (CSA). For *Roeboides* spp., which have a characteristic nuchal hump, we also measured body depth at the anterior insertion point of the anal fin (BD_A_) to get a more comprehensive body depth measurement.

### 2.6 Statistical analyses

#### 2.6.1 Overall body shape

To test for variation in body morphology of *A. ruberrimus* and *Roeboides* spp.*,* we performed a series of multivariate analyses of covariance (MANCOVAs) with the 24 principal component (PC) scores as response variables. We fit separate models for each species, and for each of the four comparisons listed in Table 2. For the impoundment effect (a), we tested for variation between habitats (streams vs. reservoir) and among sites (nested within habitat). For the invasion effect through time (b), we tested for variation between sites (Gatun, Chagres) and through time (pre vs. postinvasion). For the invasion effect across space (c), we examined variation across sites (Gatun vs. Bayano). Finally, for the temporal controls (d), we examined variation among sites and through time. Centroid size was also included in all models as a covariate. We initially included all two‐ and three‐way interactions between factors, but those that were nonsignificant were removed from the final models. Statistical significance was determined using an *F* test based on Wilks’λ. Effect size was quantified in terms of partial variance (partial η^2^, Langerhans & DeWitt, 2004).

Finally, to visualize variation in body shape across all populations independent of the potential effects of allometry, we performed MANCOVAs for each species with the shape variables (PCs) as the dependent variable and centroid size as a covariate (Franssen, 2011) and plotted the resulting residuals for PC1–PC3, which cumulatively explained 52% and 59% of shape variation for *Astyanax* and *Roeboides*, respectively (Supporting Information Table S2; Figures S1 and S2).

Visual inspection of the PCs suggested that PC1 was mainly related to the lateral bending of specimens (Supporting Information Figures S1 and S2), so we focused our interpretations on variation along PC2 and PC3.

#### 2.6.2 Linear traits

Linear traits were first standardized to a common body size, using the following allometric equation (Hendry & Taylor, 2004):

$$Z_{\text{std}}=Z_{O}{\left(\text{meanSL}/{\text{SL}}_{O}\right)}^{b}$$

where, for a given individual, *Z*_std_ is the size‐standardized trait value, *Z*_O_ is the observed trait value, SL is the mean standard length of all fish, SL_O_ is the observed standard length, and b is the common slope from a regression of log(*Z*_o_)~log(SL_O_) + population for each trait. Before fitting a common slope, we tested for heterogeneity of slopes across populations. We found significant interactions between population and standard length for 4 out of 6 traits for *A. ruberrimus*, and 2 out of 6 traits for *Roeboides* spp.; however, visual inspection of the data showed that slopes were very similar, so we proceeded to fit a common slope. All size‐standardized traits were no longer correlated with standard length (*p* > 0.05).

Second, we performed principal components analyses (PCAs) on size‐standardized traits separately for each species to visualize how populations grouped in multivariate trait space. PC1 (which explained 62.2% and 80.5% of variation for *A. ruberrimus* and *Roeboides* spp., respectively) was then used as the dependent variable in subsequent analyses. For both species, PC1 related mainly to differences in body depth, with more positive PC1 scores corresponding to lower values of maximum and anterior body depth (Supporting Information Table S3).

Third, we performed two separate ANOVAs examining variation in PC1 for each species across all populations. Post hoc planned contrasts were then used to determine the significance of particular comparisons corresponding to our questions of interest (Table 2).

Fourth, we used linear discriminant analysis (LDA) on size‐standardized traits to explore how well populations of each species could be discriminated based on their phenotypes. Classification success in the LDA was evaluated using leave‐out‐out cross‐validation implemented using the lda() function in R.

Fifth, to examine detailed patterns of trait variation, we also performed individual ANCOVAs that examined variation in each size‐standardized trait across populations within each species (Supporting Information Table S4). These were followed by planned contrasts testing our questions of interest (Table 2). All analyses were performed in R (R Core Team, 2018).

## 3 RESULTS

### 3.1 Impoundment effect

**Figure 3:**
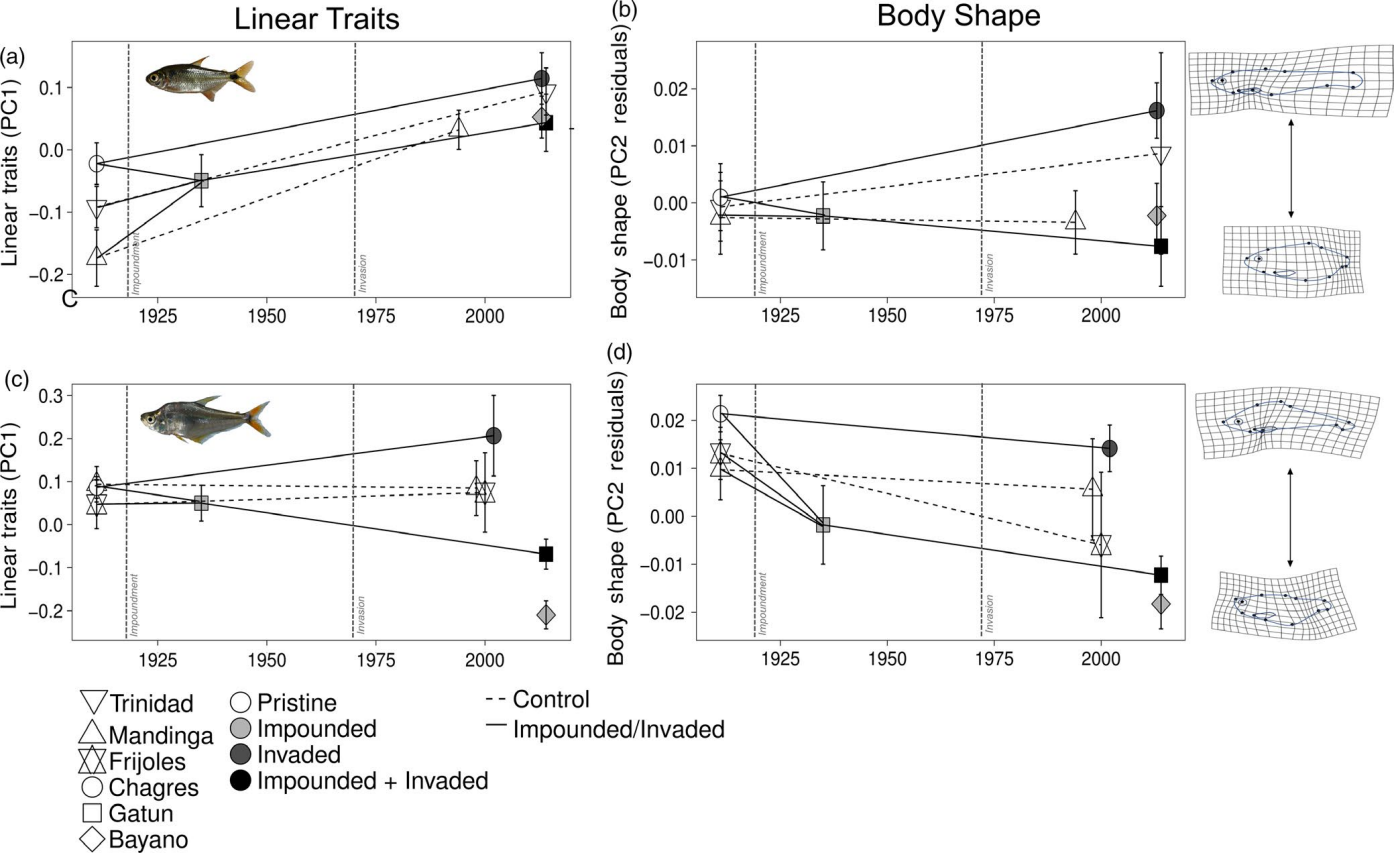
Morphological variation for *A. ruberrimus* and *Roeboides* spp. through time. Data shown are means (± 2 *SE*) of PC1 scores from a PCA on size‐adjusted traits for linear traits and size‐adjusted residuals of PC2 scores for body shape. Populations are coded by habitat (squares/diamonds for lakes, circles for large rivers, and triangles for small streams), by perturbation type (white for pristine, light gray for impounded, dark gray for invaded, and black for impounded +invaded), and by site classification (dotted line for control and solid line for impact). Lines were drawn between endpoints to facilitate the visualization of temporal trends, but should be interpreted with caution, given that traits were not sampled continuously through time, as so the actual shape of the trend is unknown. A visual representation of the extreme points of PC2 residuals for the body shape results is shown to the right of the time series. Shape deformations are shown in reference to the mean shape and have been magnified by a factor of 3

**Figure 4:**
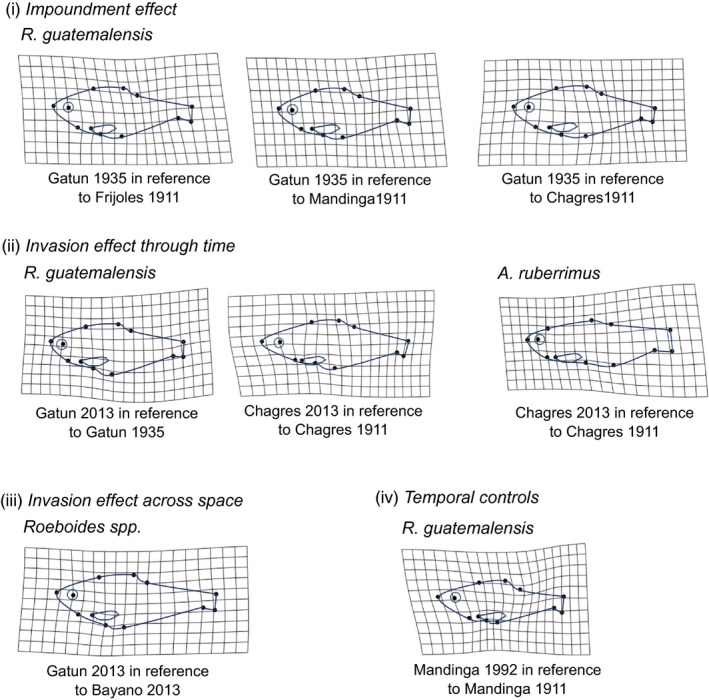
A visual representation of the statistically significant geometric morphometric results for *Astyanax ruberrimus* and *Roeboides* spp. Each population is plotted in reference to another (as labeled), distorting the grid where they differ. The distortion has been magnified by a factor of 3

The morphology of *A. ruberrimus* in the newly impounded Lake Gatun (1935) was generally overlapping with, and intermediate between, the morphology of the three source stream populations (Trinidad 1911, Mandinga 1911, Chagres 1911, Figure 3). Comparisons between these riverine source populations and the recently impounded Lake Gatun revealed no differences either in overall body shape (Table 4, Figure 3b), or for PC1 of linear traits (Table 5, Figure 3a). Separate analysis of individual traits, however, suggested a decrease in eye area (15.4%) and increase in caudal spot size (16%–19%) following impoundment (Supporting Information Figure S3, Table S4, Table S5). For *R. guatemalensis*, the same comparisons suggested there was a change in overall body shape following impoundment (Table 4, Figure 4). Specifically, *R. guatemalensis* from the newly impounded Lake Gatun had smaller heads, deeper, fuller mid‐bodies, shorter anal fins, more upturned mouths, a greater distance between the pelvic and anal fins, and deeper caudal regions (i.e., lower scores along PC2; Figure 3d and S2, Table 1). However, more variance was explained by allometry (centroid size) than by habitat (stream vs. reservoir; Table 4), and linear traits only showed an increase in the Gatun 1935 population in caudal spot area (14.5%) in comparison with Frijoles 1911 and an increase in eye area (12.43%) in comparison with Chagres 1911 (Table 5, Figure 3c, Supporting Information Table S4; Table S5).

**Table 4:** Multivariate analysis of covariance (MANCOVA) examining variation in overall body shape of *A. ruberrimus* and *Roeboides* spp

Effect	Taxa	Factor	*F*	*df*	*p*	Partial variance
(a) Impoundment	*A. ruberrimus*	Habitat	0.721	48, 208	0.911	0.143
		Population(Habitat)	0.700	24, 104	0.842	0.139
		CS	1.057	24, 104	0.404	0.196
	*R. guatemalensis*	**Habitat**	**4.766**	**48, 160**	**<0.0001**	**0.588**
		Population(Habitat)	1.208	24, 80	0.261	0.266
		**CS**	**8.094**	**24, 80**	**<0.0001**	**0.708**
(b) Invasion through time	*A. ruberrimus*	Site	1.143	24, 106	0.313	0.206
		**Time**	**1.901**	**24, 106**	**0.014**	**0.301**
		**Site × Time**	**2.036**	**24, 106**	**0.007**	**0.315**
		CS	1.129	24, 106	0.327	0.204
	*R. guatemalensis*	**Site**	**5.477**	**24, 78**	**<0.0001**	**0.628**
		**Time**	**3.478**	**24, 78**	**<0.0001**	**0.517**
		**Site × Time**	**4.752**	**24, 78**	**<0.0001**	**0.594**
		**CS**	**3.923**	**24, 78**	**<0.0001**	**0.547**
(c) Invasion across space	*A. ruberrimus*	Lake	0.661	24, 53	0.865	0.230
		CS	1.419	24, 53	0.144	0.391
	*Roeboides* spp.	**Lake**	**3.172**	**24, 58**	**0.000**	**0.568**
		**CS**	**3.187**	**24, 58**	**0.000**	**0.569**
		**Lake × CS**	**1.813**	**24, 58**	**0.034**	**0.429**
(d) Temporal controls	*A. ruberrimus*	Site	0.932	24, 76	0.561	0.227
		Time	0.809	24, 76	0.715	0.203
		CS	1.446	24, 76	0.116	0.313
	*R. guatemalensis*	Site	2.712	24, 36	0.003	0.644
		**Time**	**12.262**	**24, 36**	**<0.0001**	**0.891**
		Site ×Time	3.425	24, 36	0.000	0.695
		**CS**	**5.439**	**24, 36**	**<0.0001**	**0.784**

Statistically significant (p<0.05) results are in bold

**Table 5:** Results of planned contrasts comparing specific population means (or groups of means) in order to address our questions of interest (Table 2)

Test	Taxa	Contrast	*df*	T	*p*
(a) Impoundment effect	*Astyanax*	Streams versus Gatun 1935	242	−1.927	0.203
	*Roeboides*	Streams versus Gatun 1935	157	0.978	0.798
(b) Invasion through time	***Astyanax***	**Gatun and Chagres, pre** versus **post**	**242**	**−5.663**	**<0.001**
	*Roeboides*	Gatun and Chagres, pre versus post	157	−0.098	1.000
(c) Invasion across space	*Astyanax*	Gatun 2013 versus Bayano 2013	242	−0.214	0.999
	***Roeboides***	**Gatun 2013** versus** Bayano 2013**	**157**	**5.180**	**<0.001**
(d) Temporal controls	***Astyanax***	**Streams, pre** versus **post**	**242**	**−9.048**	**<0.001**
	*Roeboides*	Streams, pre versus post	157	−0.290	0.997

Statistically significant (p<0.05) results are in bold

### 3.2 Invasion effect through time

For *A. ruberrimus*, the response of body shape to invasion was site‐specific (Table 4). Overall body shape did not differ between specimens collected before (1935) versus after (2014) the introduction of peacock bass into Lake Gatun (Figure 3b). In contrast, in the Chagres River, overall body shape differed pre (1911) versus postinvasion (2013) (Figure 3b), with the contemporary (postinvasion) Chagres population having smaller heads, shallower mid‐bodies, and larger, deeper caudal peduncle regions (Figure 4, Table 1). In contrast, no change was evident in body shape of the two control populations (Trinidad and Mandinga streams) over the same time period (Table 4; Figure 3b). Linear traits changed significantly in all populations over time (Table 5, S4, S5); however, these changes were largely parallel between control and invaded populations (Figure 3a). All populations showed an increase in PC1 scores over time, which was driven primarily by a decrease in anterior (6%–7%) and maximum (7%) body depth over time (Supporting Information Figure S3, Tables S3–S5).

For *R. guatemalensis,* we also observed divergent morphological responses in the two invaded populations (Lake Gatun and Chagres River) over time (Table 4, Figure 3). For overall body shape, postintroduction specimens from Lake Gatun tended to have deeper bodies, a smaller anterior region, and a larger, deeper caudal peduncle area relative to preintroduction specimens (Figure 4; Table 1 and Table S4). However, in the Chagres River, *R. guatemalensis* shifted toward a longer anterior region, shallower mid‐bodies, and smaller caudal peduncle regions postintroduction (Figure 4; Table 1 and Supporting Information Table S4). Change in overall body shape was also observed over time in control populations (Mandinga and Frijoles) (Table 4) but was driven by the Mandinga population which showed shallower bodies and caudal peduncle region over time (Figure 4). A similarly divergent pattern in invaded populations was observed for linear traits with lower PC1 scores (i.e., deeper bodies; Figure 3c, S4, Supporting Information Table S3) observed postintroduction for Gatun, but not Chagres; however, no overall effect of invasion was evident when temporal trends for the two populations were analyzed together (Table 5, Figure 3c). Caudal spot size increased (27.7%) postinvasion in Gatun, but not in Chagres (Supporting Information Tables S4 and S5). Linear traits did not change over time in the two control streams (Table 5).

### 3.3 Invasion effect across space

For *A. ruberrimus*, MANCOVA on geometric morphometric variables showed no overall difference in body morphology between populations from the invaded Lake Gatun and the uninvaded Lake Bayano (Table 4). Similarly, no difference was evident for linear traits between these populations (Table 5, Figure 3a, Supporting Information Tables S4 and S5). For *Roeboides* spp., MANCOVA on geometric morphometric variables revealed a difference in overall body shape between the invaded Lake Gatun and the uninvaded Lake Bayano (Table 4). Specifically, individuals from Lake Gatun had smaller anterior regions and shallower bodies (Figure 4; Supporting Information Table S4; lower scores along PC3; Supporting Information Figure S2). Linear traits also differed between these populations (Table 5), with fish from Bayano having lower PC1 scores, that is, deeper bodies (4%–5%), smaller anterior regions (4.81%), and smaller caudal peduncle depths (5.58%) (Figure 3 c, Supporting Information Tables S3–Table S5).

### 3.4 Population discrimination in multivariate space

**Figure 5:**
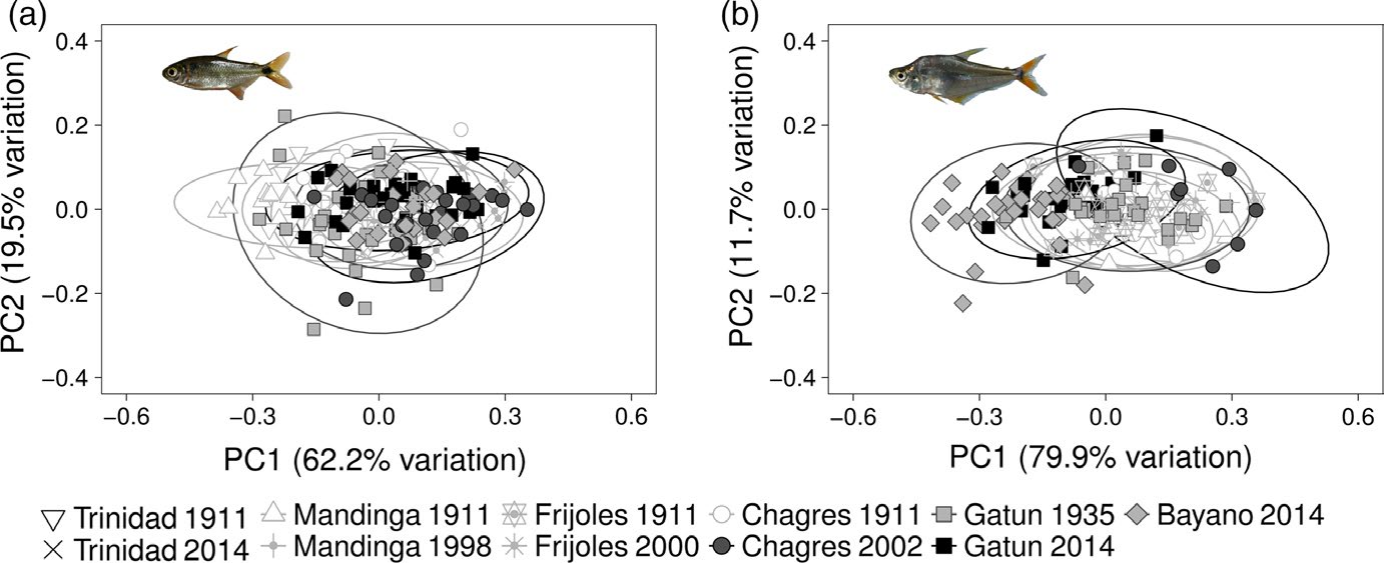
Principal components analysis (PCA) of linear traits for *Astyanax ruberrimus* (panel A) and *Roeboides* spp. (panel B). Populations are coded by habitat (squares/diamonds for lakes, circles for large rivers, and triangles/stars for small streams) and by perturbation type (light gray fill for unperturbed, dark gray for impounded but not invaded, and black for impounded and invaded). Ellipses probability is set at 95%. Trait loadings may be referred to in Table S3

For *A. ruberrimus,* PCA and LDA visualizations showed that all populations overlapped extensively in multivariate trait space (Figure 5 a and S5). Indeed, within each population, on average only 52.8% of individuals (range 30.0%–72.7%) were correctly assigned to their own population, not much better than by chance alone (50%). Similarly, for *Roeboides* spp., LDA and PCA plots revealed large overlap among populations in multivariate trait space (Figure 5 b and S6), and individuals could be correctly assigned to their population of origin only 58.7% of the time.

## 4 DISCUSSION

The increased prevalence of anthropogenic stressors makes it important to understand the (in)ability of species to respond to novel selection regimes. We tested for evidence of morphological change in two native fishes (*Astyanax ruberrimus* and *Roeboides* spp.) following the impoundment and subsequent invasion by a piscivorous predator into Lake Gatun, but found only limited evidence of change, as we outline below.

### 4.1 Impoundment effect

Overall, the morphological response to impoundment was taxon‐specific. In *A. ruberrimus*, no change was evident in either body shape or linear traits (PC1). However, when linear traits were analyzed separately, we did observe an increase in caudal spot size and decrease in eye area postimpoundment. Caudal markings (spots, ocelli) appear to have an antipredator function in many fishes. For example, caudal ocelli reduce the incidence of cannibalism in *Cichla monoculus* (Zaret, 1977), and of fin predation in *Astronotus ocellatus* (Winemiller, 1990). In many prey fish, the combination of a smaller eye and larger caudal spot has been shown to divert predator strikes away from the head, thus increasing the probability of escape in an encounter with a predator (Carroll, Wainwright, Huskey, Collar, & Turingan, 2004; Kjernsmo & Merilaita, 2013; Lönnstedt, McCormick, & Chivers, 2013; McPhail, 1977). One possible explanation for the decrease in eye size and increase in caudal spot size in *Astyanax* following impoundment is thus that predation was higher in the newly formed Lake Gatun than in the rivers and streams that preceded it. This change could have happened if, for example, native predators (e.g., *Hoplias microlepis*) increased in either abundance or body size following impoundment, which often occurs in newly formed reservoirs (Franssen, 2011). Alternatively (or jointly), there could have been a change in water clarity and the light environment following impoundment that altered selection on both eyes and visual signals (e.g., spots). However, given the uncertainty in historical environmental and ecological conditions, it is difficult to interpret the potential adaptive significance of historical phenotypes.

In *R. guatemalensis*, overall body shape did shift postimpoundment, with fish from the newly impounded Lake Gatun having smaller heads, deeper, fuller mid‐bodies, shorter anal fins, more upturned mouths, a greater distance between the pelvic and anal fins, and deeper caudal regions than individuals from the three source rivers. These findings parallel some of the morphological shifts observed in temperate cyprinids following impoundment (e.g., *Cyprinella venusta*, Haas, Blum, & Heins, 2010; *Cyprinella lutrensis*, Franssen, 2011) and may reflect divergent selection in lotic versus lentic environments.

The highly divergent responses to impoundment in *A. ruberrimus* compared to *R. guatemalensis* when simultaneously subjected to the same pressures may potentially be explained by differences in initial body shape and ecology. In their native riverine habitats, *Astyanax* are typically found at the surface or in the middle of the water column. They have a tendency to school and can be very active, even aggressive (Angermeier & Karr, 1983; Breder, 1943; Zaret & Rand, 1971). *Astyanax* have moderately elongate and oval‐shaped bodies typical of many fishes (*i.e., Hemigrammus* and *Hyphessobrycon*) that are generally adapted to a variety of ecological contexts (Helfman, Collette, Facey, & Bowen, 2009; de Melo & Buckup, 2006). In contrast, *Roeboides* are relatively benthic, inhabiting quiet, slow‐moving creeks, where they feed on fish scales and benthic invertebrates (Bussing, 2002; Kramer & Bryant, 1995). They have a very atypical (hump‐shaped) body form and a compressed, almost transparent body with very little muscle mass in the caudal region (Bussing, 2002). We propose that *A. ruberrimus* may have been more pre‐adapted to lake habitats than *R. guatemalensis,* resulting in stronger selection pressures acting on the latter (Hendry et al., 2002; Sharpe et al., 2017; Storfer, 1999).

In general, much remains unknown about morphological responses to impoundment. Studies examining morphological variation between naturally occurring river and lake fish populations have shown responses to be highly variable across families. For example, lentic populations were deeper‐bodied than their lotic counterparts in Cichlidae and Characidae (Langerhans, 2008; Langerhans, Layman, Langerhans, & Dewitt, 2003), but were shallower‐bodied in Gasterosteidae (Hendry et al., 2002; Sharpe, Räsänen, Berner, & Hendry, 2008) and Salmonidae (Pakkasmaa & Piironen, 2001). Similar contrasting patterns across taxa have been observed for caudal peduncle area and depth (Krabbenhoft, Collyer, & Quattro, 2009; Langerhans et al., 2003). To our knowledge, only a few studies to date have explicitly looked at the morphological effects of impoundment. Paralleling our results for *Roeboides* (but not *Astyanax*), all found substantial, parallel morphological shifts (a decrease in head size, and an increase in body depth) in reservoir versus stream populations (Franssen, 2011; Franssen et al., 2012; Franssen, Stewart, & Schaefer, 2013; Haas et al., 2010). The direction and magnitude of any change in body shape may ultimately be dependent on the habitat the species colonizes in the newly formed reservoir. For example, if a population colonizes the littoral zone where the habitat is more complex, this might select for a body shape that enhances maneuverability (i.e., deeper body) (Langerhans & Reznick, 2010). In contrast, if it colonizes the pelagic zone, a decrease in body depth might be more advantageous (Sharpe et al., 2008; Walker, 1997). It must also be noted that sex (Kitano, Mori, & Peichel, 2007), reproductive condition (Plaut, 2002), and diet (Meyer, 1989) have been shown to influence body shape in other fishes. Although we were not able to directly asses these factors in the current study, we assume that our random sampling included an even distribution of individuals of both sexes, and therefore that they did not generate any systematic biases in our results.

### 4.2 Invasion effect

Morphological responses to invasion varied in both direction and magnitude among species, sites, and comparisons (time vs. space). A well‐developed literature on phenotypic responses to predation helps to interpret the observed variation (reviewed in Agrawal, 2001; Benard, 2004; Langerhans & Reznick, 2010). Predictions include less streamlined body shapes characterized by smaller heads and anterior regions but larger/deeper mid‐body and caudal regions in high‐predation environments to possibly misdirect strikes (Webb, 1986), increase performance in escape maneuvers (Langerhans & Reznick, 2010; Law & Blake, 1996), and deter gape‐limited predators (Domenici, Turesson, Brodersen, & Brönmark, 2008; Langerhans & DeWitt, 2004; Langerhans, Layman, Shokrollahi, DeWitt, & Wainwright, 2004; Langerhans & Reznick, 2010). As outlined above, caudal spot size also might be expected to increase in high‐predation contexts (Lönnstedt et al., 2013).

In *A. ruberrimus,* no detectable change was found in body shape after the peacock bass introduction in Lake Gatun, either over time or across space. In contrast, in the Chagres River population, the postinvasion population had smaller heads (as predicted), shallower bodies (contrary to predictions), and increased caudal peduncle sizes (as predicted). In *R. guatemalensis*, the Gatun population showed a decrease in head size and an increase in mid‐body and caudal depth postinvasion (as predicted). In contrast, the Chagres population had longer heads, shallower bodies, and smaller caudal regions postinvasion (contrary to predictions). In the spatial comparison, body depth was greater in *R. occidentalis* in the uninvaded Lake Bayano (contrary to predictions). However, comparisons of *Roeboides* spp. between Lake Gatun and Lake Bayano must be interpreted with caution, as they have been described as separate species (Meek & Hildebrand, 1916). We felt this comparison was still worth including given the similarity between species (Meek & Hildebrand, 1916), although we are not able to distinguish between different hypotheses (are the phenotypic differences between these populations the result of contemporary adaptation to divergent predation regimes, or the result of drift following spatial isolation?).

It is interesting that both species showed divergent responses between the invaded river (Chagres) and lake (Gatun) sites and that both shifted toward shallower bodies in the former. It is possible that differences in flow regime are exerting different selection pressures and perhaps selecting for shallower, more streamlined body forms in the fast‐flowing Chagres River relative to Lake Gatun (Brinsmead & Fox, 2002; Langerhans et al., 2003; Pakkasmaa & Piironen, 2001).

### 4.3 Does limited and contrasting morphological change imply maladaptation?

We envisioned three possible scenarios for how our study species might respond to impoundment and subsequent invasion. The first (parallel and significant morphological change) and the second (no morphological change) were both not unequivocally supported by our data. The third scenario (that species would show subtle and/or contrasting morphological changes) is what we observed in the majority of cases. However, do these complex patterns imply populations are maladapted?

Maladaptation is often inferred when traits deviate from some idealized “optimum.” However, there are a number of limitations with this logic. First, phenotypic optima are often inferred from biomechanical principles (e.g., Langerhans, 2008; Langerhans & Reznick, 2010); however, in practice, these generalizations may not apply equally across taxa. Second, there can often be multiple adaptive solutions to a given ecological problem, resulting in multiple optima. For example, in heterogeneous environments, selection could favor either the evolution of divergent specialist phenotypes or a single generalist form (Tienderen, 1991). In our study, we proposed that the more generalist morphology of *A. ruberrimus* was pre‐adapted to the lentic environment to a greater degree than the specialized body shape of *Roeboides* spp., perhaps explaining their divergent responses to impoundment.

Third, optima may be difficult to define (or achieve) when multiple selective factors interact (McBryan, Anttila, Healy, & Schulte, 2013; Schulte, 2007). Thus, deviations from “expected” phenotypes could reflect maladaptation, but could also reflect local adaptation to site factors (Stuart et al., 2017). Indeed, nonparallel responses arising from complex interactions between factors are quite common in nature (Oke, Rolshausen, LeBlond, & Hendry, 2017). In our study, we hypothesized that nonparallel morphological responses to invasion between Lake Gatun and the Chagres River might reflect conflicting selective pressures. Specifically, the body shape of both *A. ruberrimus* and *R. guatemalensis* in the Chagres population could reflect a compromise between predator escape and drag reduction in a high‐flow environment.

Fourth, phenotypes may deviate from expected values simply because populations are instead responding in other (nonmorphological) ways. For example, riverine fish may successfully persist in reservoirs by occupying the littoral zone where the habitat is most similar to that of rivers and streams (Agostinho, Gomes, Santos, Ortega, & Pelicice, 2016; Gillette, Tiemann, Edds, & Wildhaber, 2005). Prey may respond to introduced predators through shifts in life history traits rather than morphology (Sharpe et al., 2015; Sharpe, Wandera, & Chapman, 2012). Other strategies for adapting to a novel predator include the adoption of antipredator behaviors such as shoaling. This strategy increases vigilance and predator recognition and allows for a variety of response tactics, including confusing the predator when attacked (Magurran, 1990). This possibility is especially pertinent to *A. ruberrimus*, which is known to be a shoaling species (Zaret, 1984). In fact, we have found that *A. ruberrimus* adjust their shoaling behavior in response to chemical alarm cues from predators (Sharpe et al., *in prep*). Other behavioral responses include switching habitats, for example, reducing the use of open habitats in the presence of predators (Werner et al., 1983), decreasing activity levels to reduce conspicuousness, and shifting activities to other times of the day when predators are less successful (Reebs, 2008). We have observed that populations of both species from Lake Gatun are often found hiding in vegetation and are much more difficult to catch than their counterparts from the streams (pers. obs.). *Roeboides* spp. are known to be nocturnal (Zaret, 1984), which is a strategy that could limit predation by *C. monoculus*, a visual predator that hunts by day. Overall, if *A. ruberrimus* and *Roeboides* spp. have managed to partially mitigate the impacts of impoundment and invasion through a combination of habitat choice, altered life history strategies, and/or adaptive behavior, then this might weaken selection on morphology.

Fifth, phenotypic change may be difficult to detect if insufficient time has passed for evolution to occur. Assuming a generation time of two years for *Astyanax* (Fumey et al., 2018) and three years for *Roeboides* (Winemiller, 1989), we estimate that our entire time series (1911–2013) corresponded to roughly 51 generations for the former and 34 generations for the latter. In theory, this should be long enough to observe an evolutionary response, but only if selection were strong and consistent, and acting on the traits in question (see above).

Ultimately, (mal)adaptation cannot be inferred from trait‐based approaches alone. Reciprocal transplants and/or quantification of fitness correlates are required to understand the functional significance and fitness consequences of observed morphological patterns. Another very important distinction is the difference between absolute and relative maladaptation (Hendry & Gonzalez, 2008). A population could exhibit relative maladaptation (i.e., have a lower fitness than some idealized reference value), but still not show absolute maladaptation (a negative growth rate). This scenario likely applies to *A. ruberrimus* and *Roeboides* spp., which both declined drastically in abundance after the peacock bass introduction (Zaret & Paine, 1973), yet do still persist 45 years later, albeit at very low densities (Sharpe et al., 2017).

## 5 CONCLUSION

The current rate of human‐induced environmental change has led to a biodiversity crisis (Wood et al., 2000), in some cases challenging species to either adapt or disappear. It has thus become of great interest to integrate ecological and evolutionary responses to make reliable predictions as to the ability of species to adapt to novel stressors. Our study shows that morphological responses to multiple stressors can be very limited in some cases and, when they do occur, are often complex and context‐dependent.

The increasing prevalence of dams (Zarfl, Lumsdon, Berlekamp, Tydecks, & Tockner, 2015) and biological invasions (Hall & Mills, 2000; Lodge, 1993) in the tropics (Turgeon et al., 2018) requires a better understanding of how multiple stressors might interact and affect native species. A special focus is needed in understudied tropical ecosystems which have a unique evolutionary history and host a uniquely diverse range of species.

## CONFLICT OF INTEREST

None declared.

## Data Availability

Data will be archived in the Dryad Digital Repository (to be completed after manuscript is accepted for publication). https://doi.org/10.5061/dryad.4tb7301.
