# Supplementary figures and images for: 100‐year time series reveal little morphological change following impoundment and predator invasion in two Neotropical characids
Source: Evol Appl. 2019 Feb 27;12(7):1385–401. doi: 10.1111/eva.12763 (PMC6691216; doi:10.1111/eva.12763)

Body Shape (PC1 Residuals)

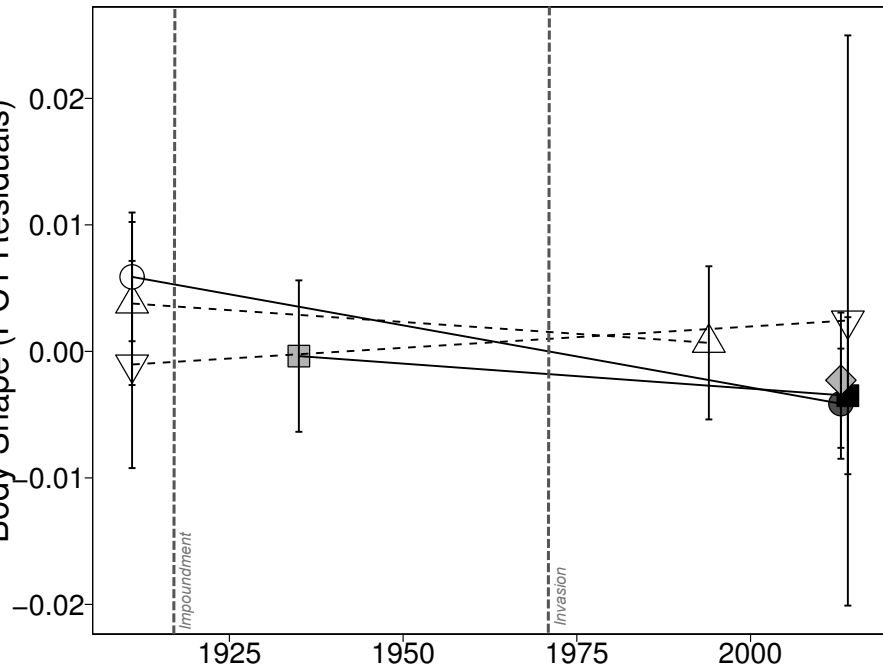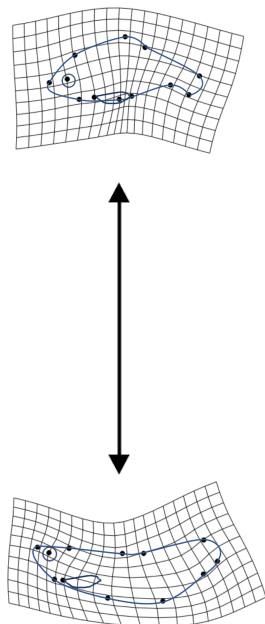

Body Shape (PC2 Residuals)

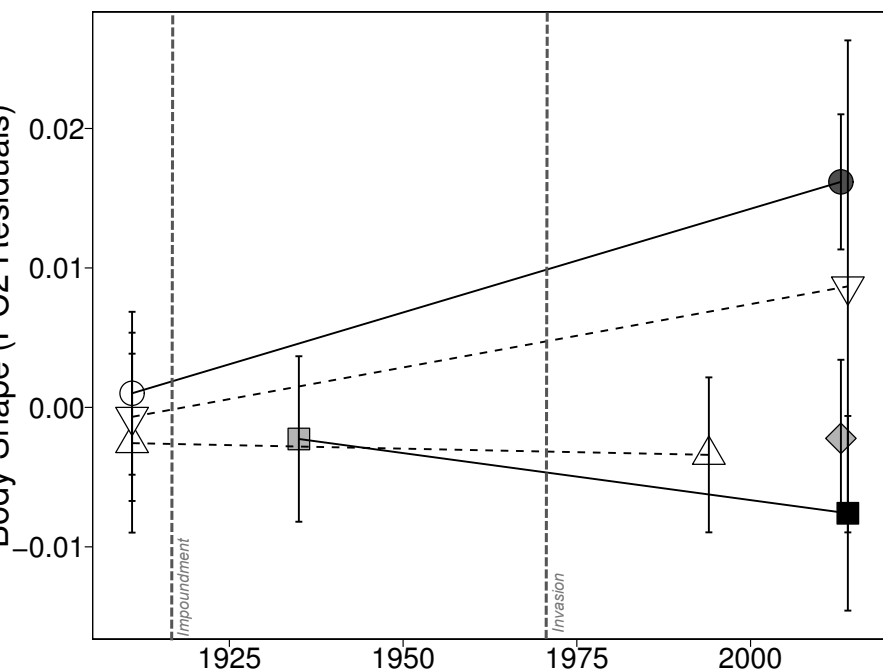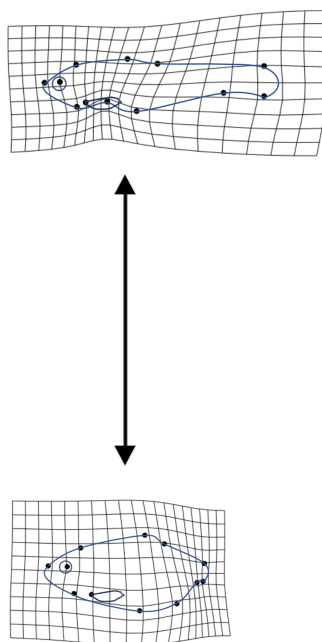

Body Shape (PC3 Residuals)

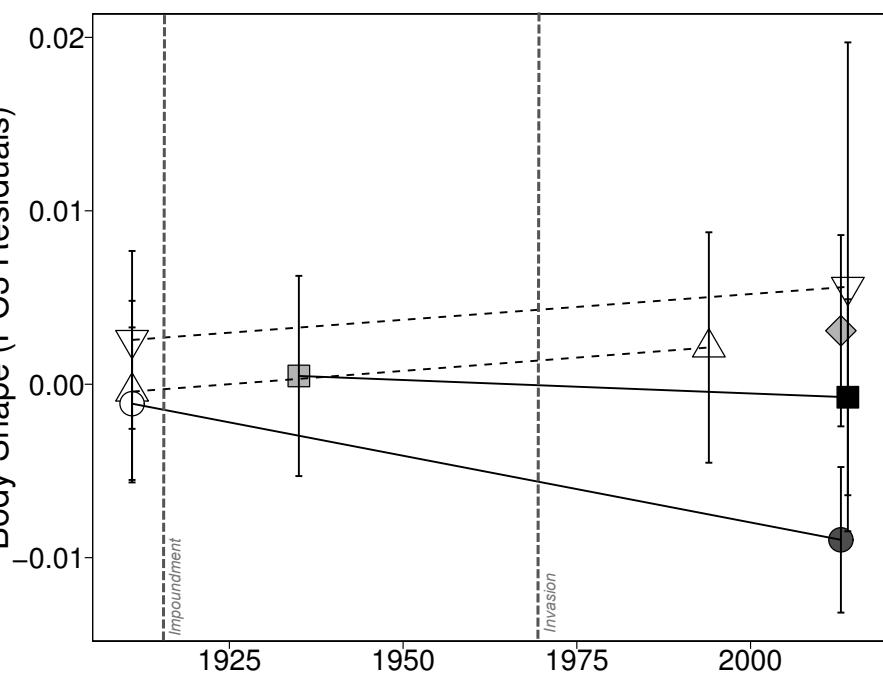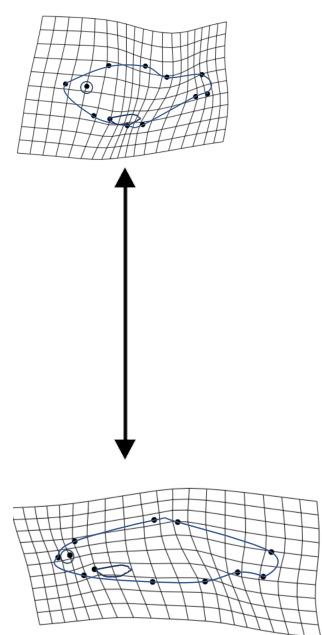

Supplement: Supplementary file 1 [file EVA-12-1385-s001.pdf]

Body Shape (PC1 Residuals)

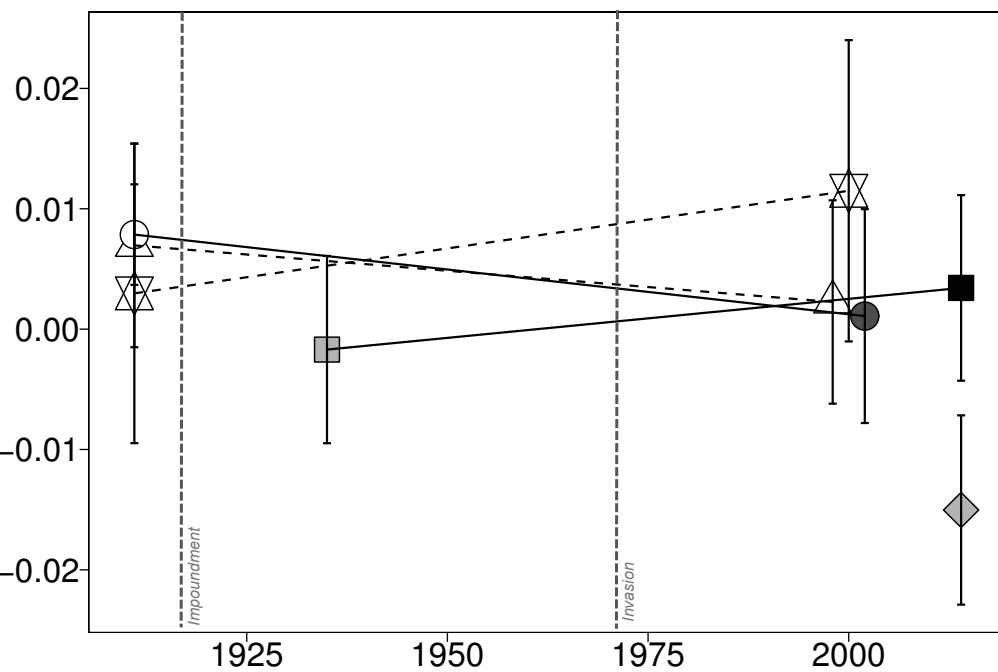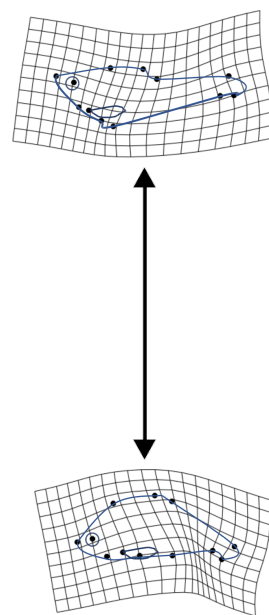

Body Shape (PC2 Residuals)

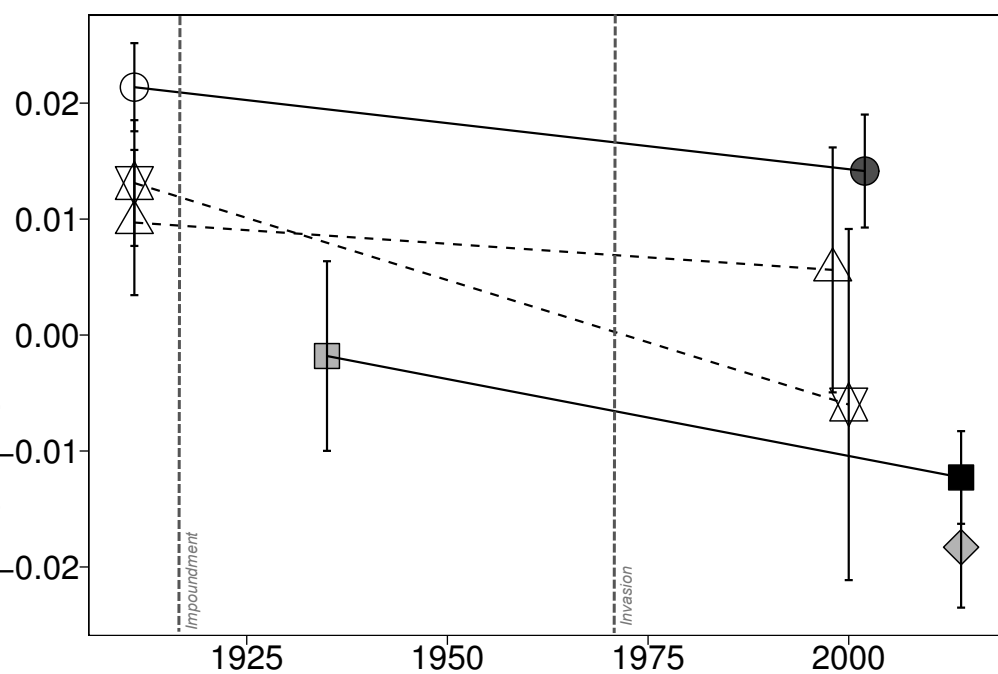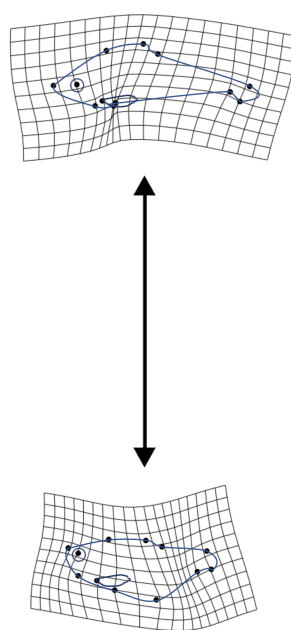

Body Shape (PC3 Residuals)

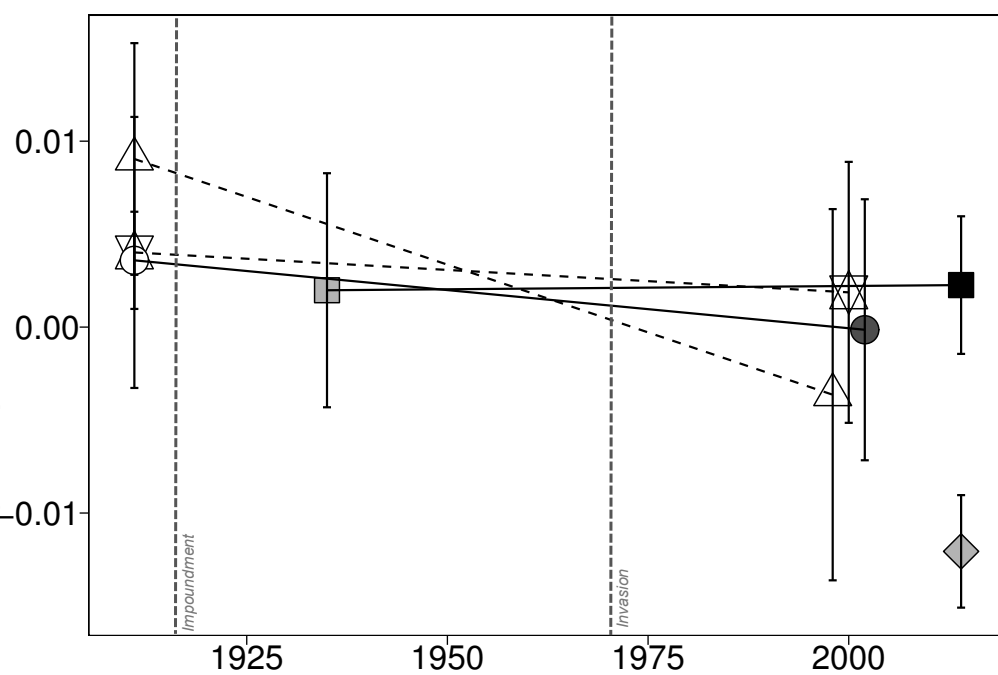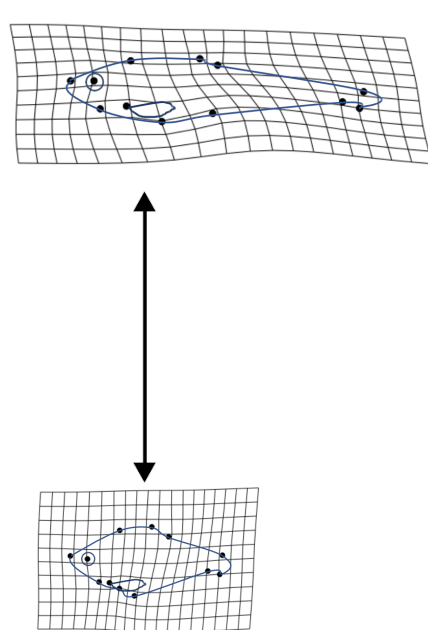

Supplement: Supplementary file 2 [file EVA-12-1385-s002.pdf]

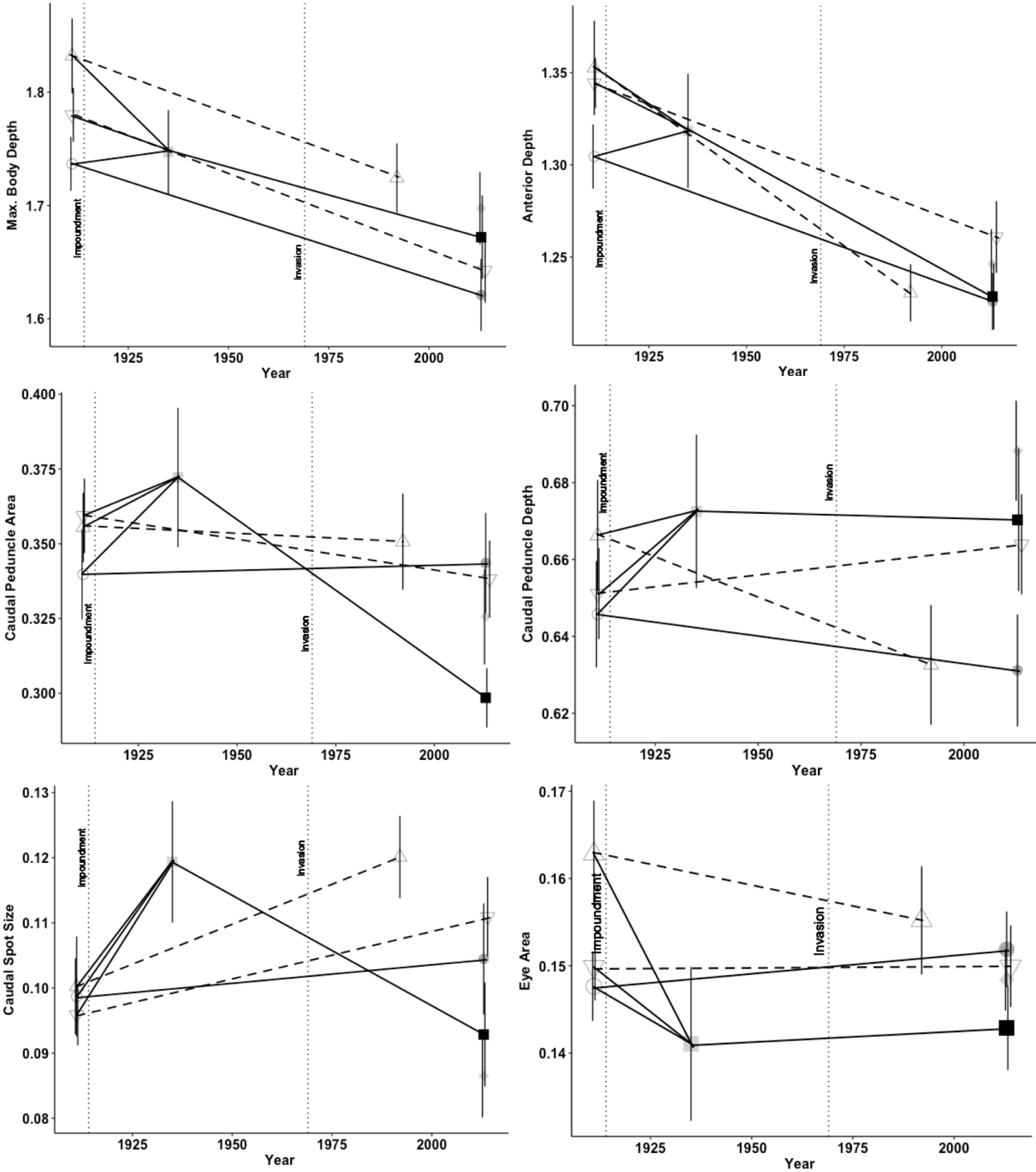

■ Gatun 1935    ◆ Bayano 2013    ● Chagres 2013    △ Mandinga 1992    ▽ Trinidad 2014  
 ■ Gatun 2013    ○ Chagres 1911    △ Mandinga 1911    ▽ Trinidad 1911

Supplement: Supplementary file 3 [file EVA-12-1385-s003.pdf]

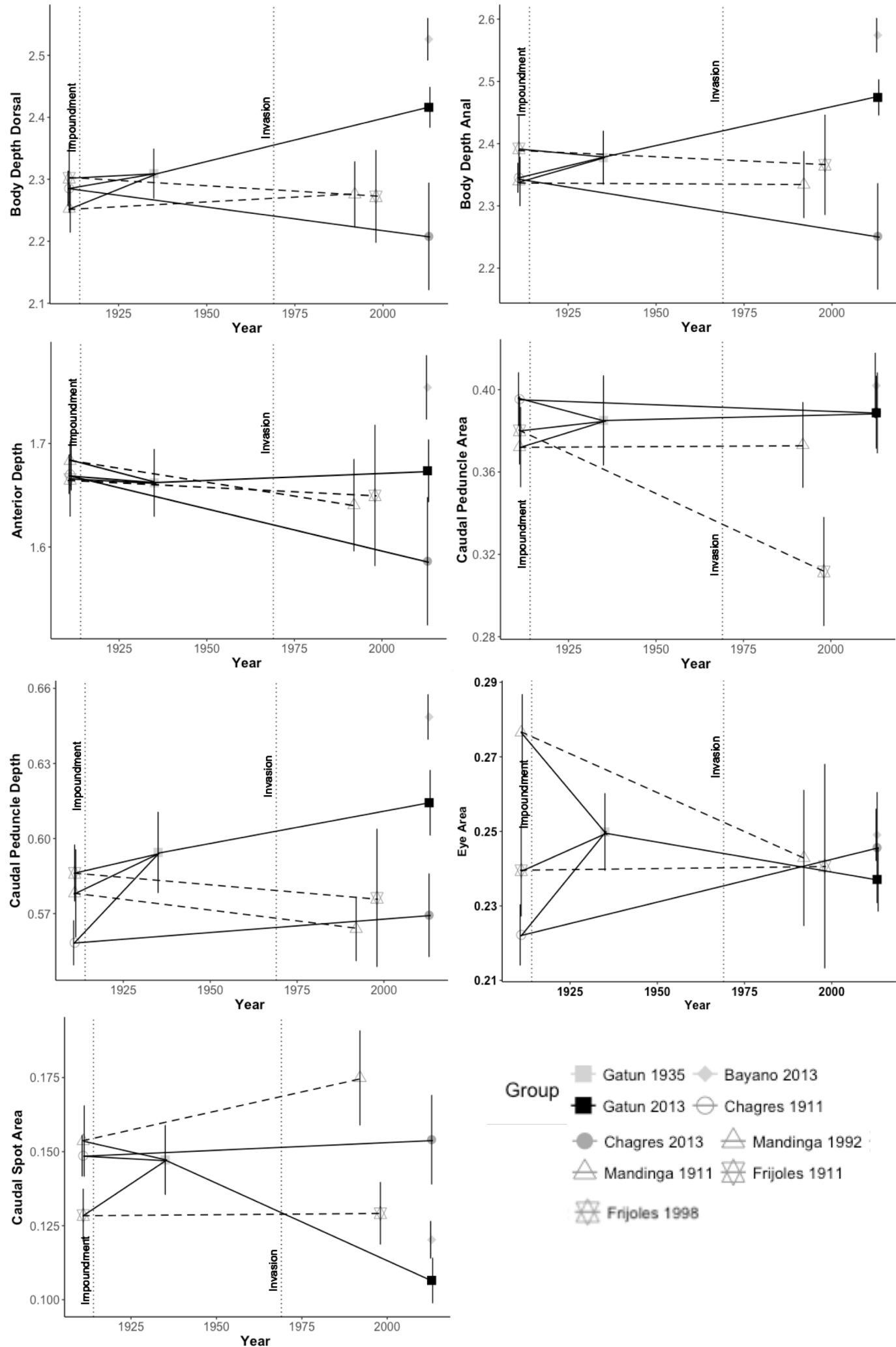

Supplement: Supplementary file 4 [file EVA-12-1385-s004.pdf]

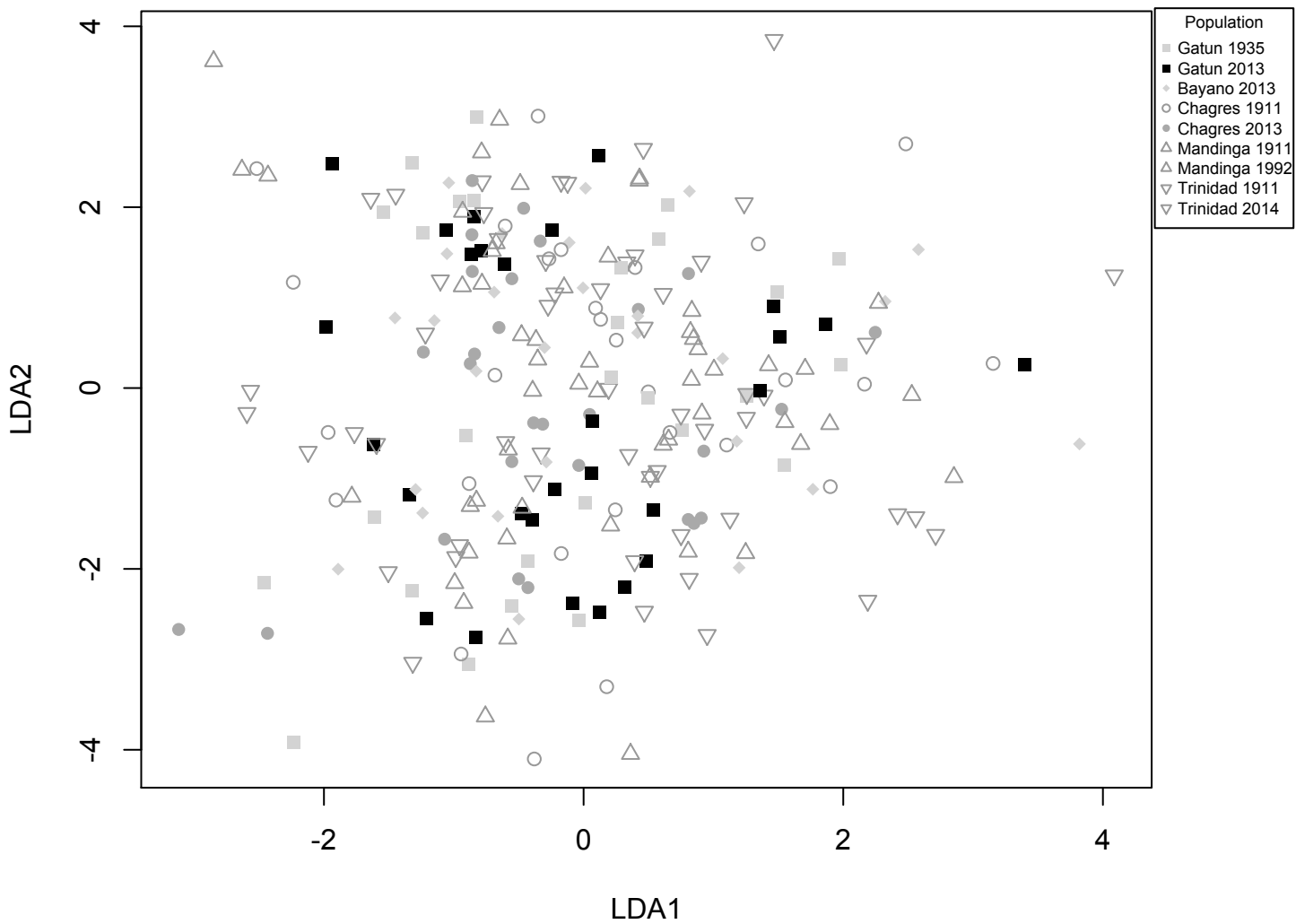

Supplement: Supplementary file 5 [file EVA-12-1385-s005.pdf]

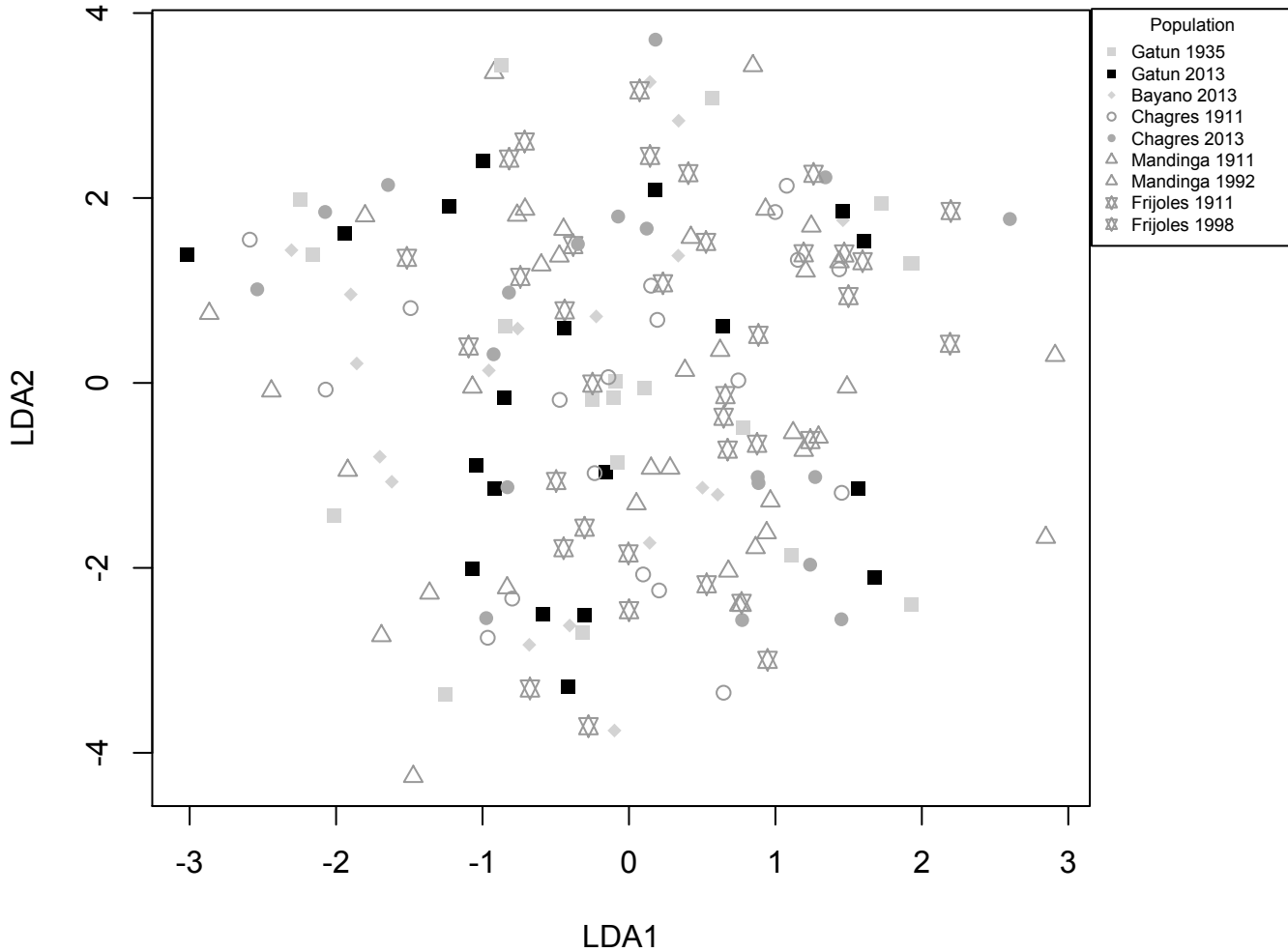

Supplement: Supplementary file 6 [file EVA-12-1385-s006.pdf]
