# Supplementary material for: 100‐year time series reveal little morphological change following impoundment and predator invasion in two Neotropical characids
Source: Evol Appl. 2019 Feb 27;12(7):1385–401. doi: 10.1111/eva.12763 (PMC6691216; doi:10.1111/eva.12763)
Supplement: Supplementary file 7 [file EVA-12-1385-s007.docx]

**Supplementary Tables**

**Table S1.** Summary of populations included in the study. Year of collection, specific collection sites and methods are indicated as recorded by the collector. Where NMNH: National Museum of Natural History; STRI: Smithsonian Tropical Research Institute, BCI: Barro Colorado Island. N/A means information is not available.

| **Species** | **Water Body** | **n** | **Year(s) collected** | **Status** | **Collectors; Source of Sample** | **Collection Site (Lat, Lon)** |
| --- | --- | --- | --- | --- | --- | --- |
| *Astyanax ruberrimus* | Gatun | 31 | 1935 | Impounded | Loftin et al; NMNH | (9.1172; -79.6923) |
|  |  | 30 | 2013 | Impounded + Invaded | Sharpe *et al.* | (9.1233, -79.7815); (9.1035, -80.0279); (9.1819, -79.8304) |
|  | Bayano | 25 | 2013 | Impounded | Sharpe *et al.* | (9.1802; -78.8843) |
|  | Chagres | 33 | 1911 | Pristine | Meek & Hildebrand;  NMNH | N/A |
|  | Mandinga | 32 | 1911 | Pristine | Meek & Hildebrand;  NMNH | Bas Obispo |
|  | Trinidad | 31 | 1911 | Pristine | Meek & Hildebrand;  NMNH | Upper Trinidad |
|  | Chagres | 30 | 2013 | Invaded | Sharpe *et al.* | (9.2226, -79.5466) |
|  | Trinidad | 30 | 2014 | Pristine | De Leon et al | N/A |
| *Roeboides guatemalensis* | Gatun | 30 | 1935, 1962 | Impounded | Loftin et al;  NMNH | (9.1172; -79.6923) |
|  |  | 36 | 2013-2015 | Impounded + Invaded | Sharpe *et al.* | (9.1233, -79.7815); (9.0928, -79.8127); (9.1819, -79.8304) |
|  | Chagres | 25 | 1911 | Pristine | Meek & Hildebrand;  NMNH | Gamboa |
|  | Mandinga | 15 | 1911 | Pristine | Meek & Hildebrand;  NMNH | Bas Obispo |
|  | Frijoles | 19 | 1911 | Pristine | Meek & Hildebrand;  NMNH | N/A |
|  | Chagres | 8 | 2002 | Invaded | STRI | (9.3601, -79.3223) |
|  | Mandinga | 12 | 1992 | Pristine | STRI | (9.0253, -79.6989) |
|  | Frijoles | 6 | 1993,1998,  1999, 2006 | Pristine | STRI | (9.1509, -79.7300); (9.1648, -79.7546); (9.1522, -79.7357); (9.1508, -79.7544) |
| *Roeboides occidentalis* | Bayano | 36 | 2014 | Impounded | Sharpe *et al.* | (9.1541, -78.8328) |

**Table S2.** PC Loadings for geometric morphometrics for *A. ruberrimus* and *Roeboides* spp.

| **PC axis** | **Proportion of Variance** | **Cumulative Proportion** |
| --- | --- | --- |
| 1. *ruberrimus* | | |
| PC1 | 0.1960 | 0.1960 |
| PC2 | 0.1849 | 0.3809 |
| PC3 | 0.1400 | 0.5209 |
| PC4 | 0.0960 | 0.6169 |
| PC5 | 0.0678 | 0.6847 |
| PC6 | 0.0601 | 0.7448 |
| PC7 | 0.0536 | 0.7984 |
| PC8 | 0.0388 | 0.8371 |
| PC9 | 0.0298 | 0.8670 |
| PC10 | 0.0282 | 0.8952 |
| PC11 | 0.0237 | 0.9188 |
| PC12 | 0.0203 | 0.9391 |
| PC13 | 0.0144 | 0.9535 |
| PC14 | 0.0108 | 0.9643 |
| PC15 | 0.0097 | 0.9740 |
| PC16 | 0.0080 | 0.9820 |
| PC17 | 0.0063 | 0.9883 |
| PC18 | 0.0049 | 0.9932 |
| PC19 | 0.0041 | 0.9973 |
| PC20 | 0.0028 | 1.0000 |
| PC21 | 0.0000 | 1.0000 |
| PC22 | 0.0000 | 1.0000 |
| PC23 | 0.0000 | 1.0000 |
| PC24 | 0.0000 | 1.0000 |
| *Roeboides spp.* | | |
| PC1 | 0.2618 | 0.2618 |
| PC2 | 0.2235 | 0.4853 |
| PC3 | 0.1027 | 0.5880 |
| PC4 | 0.0951 | 0.6831 |
| PC5 | 0.0626 | 0.7456 |
| PC6 | 0.0506 | 0.7962 |
| PC7 | 0.0418 | 0.8380 |
| PC8 | 0.0299 | 0.8679 |
| PC9 | 0.0256 | 0.8935 |
| PC10 | 0.0217 | 0.9152 |
| PC11 | 0.0175 | 0.9326 |
| PC12 | 0.0156 | 0.9482 |
| PC13 | 0.0107 | 0.9589 |
| PC14 | 0.0099 | 0.9688 |
| PC15 | 0.0078 | 0.9766 |
| PC16 | 0.0062 | 0.9828 |
| PC17 | 0.0056 | 0.9883 |
| PC18 | 0.0051 | 0.9935 |
| PC19 | 0.0040 | 0.9975 |
| PC20 | 0.0025 | 1.0000 |
| PC21 | 0.0000 | 1.0000 |
| PC22 | 0.0000 | 1.0000 |
| PC23 | 0.0000 | 1.0000 |
| PC24 | 0.0000 | 1.0000 |

**Table S3.** PC Loadings for size-standardized traits for *A. ruberrimus* and *Roeboides* spp.

| **Trait** | **PC1** | **PC2** | **PC3** | **PC4** | **PC5** | **PC6** | **PC7** |
| --- | --- | --- | --- | --- | --- | --- | --- |
| *Astyanax ruberrimus* | | | | | | | |
| % Variance Explained | 62.2 | 19.5 | 8.9 | 6.1 | 2.3 | 1.2 |  |
| Cumulative | 66.2 | 81.8 | 90.7 | 96.8 | 99.1 | 100.0 |  |
| BD | **-0.849** | 0.209 | 0.448 | -0.178 | 0.037 | 0.033 |  |
| AD | **-0.478** | 0.066 | -0.835 | 0.222 | -0.044 | -0.135 |  |
| CPD | -0.170 | -0.582 | 0.251 | **0.751** | -0.067 | 0.022 |  |
| CPA | -0.140 | -0.773 | -0.149 | **-0.564** | 0.203 | 0.045 |  |
| EA | -0.029 | 0.042 | -0.127 | 0.031 | -0.085 | **0.987** |  |
| CSA | -0.025 | -0.120 | 0.018 | -0.189 | **-0.972** | -0.071 |  |
| *Roeboides* spp. | | | | | | | |
| % Variance Explained | 80.5 | 11.4 | 2.7 | 1.9 | 1.5 | 1.2 | 1.5 |
| Cumulative | 80.5 | 91.9 | 94.6 | 96.5 | 98.1 | 99.3 | 100.0 |
| BD | **-0.696** | 0.210 | 0.031 | -0.487 | 0.357 | -0.211 | 0.248 |
| AD | -0.259 | **-0.956** | -0.085 | -0.053 | 0.057 | 0.053 | -0.049 |
| CPD | -0.158 | 0.000 | 0.459 | **0.633** | **0.550** | -0.162 | -0.186 |
| CPA | -0.059 | -0.070 | **0.857** | -0.195 | -0.405 | 0.187 | 0.144 |
| EA | -0.007 | -0.097 | -0.052 | 0.334 | -0.303 | **-0.604** | **0.647** |
| CSA | 0.053 | -0.029 | 0.106 | -0.246 | -0.207 | -0.703 | -0.622 |

**Table S4.** Analysis of covariance (ANCOVA) results examining changes in linear traits of *A. ruberrimus* and *Roeboides* spp. Only traits for which significant differences (P < 0.05) are shown. The effect size for each pairwise comparison was calculated as the difference between mean size-standardized trait values. Substantial (>10%) changes are highlighted in bold.

| **Test** | ***Taxa*** | **Comparison** | **Trait** | **d.f.** | **F** | **p** | **Trend in ref. to invaded pop.** |
| --- | --- | --- | --- | --- | --- | --- | --- |
| **(i) Impoundment effect** | *Astyanax* | Gatun 1935 vs. Mandinga 1911 | BD | 1,52 | 13.33 | <0.001 | 4.87% smaller |
|  |  |  | AD | 1,52 | 5.95 | 0.018 | 2.59% smaller |
|  |  |  | EA | 1,52 | 9.53 | 0.003 | **15.4% smaller** |
|  |  |  | CSA | 1,51 | 8.13 | 0.006 | **16.0% larger** |
|  |  | Gatun 1935 vs. Trinidad 1911 | BD | 1,58 | 7.20 | 0.010 | 1.89% smaller |
|  |  |  | CPA | 1,57 | 10.71 | 0.002 | 3.44% larger |
|  |  |  | CSA | 1,57 | 15.84 | <0.001 | **19.7% larger** |
|  |  | Gatun 1935 vs. Chagres 1911 | CPD | 1,57 | 4.26 | 0.044 | 3.98% larger |
|  |  |  | CPA | 1,57 | 12.06 | 0.001 | 8.70% larger |
|  |  |  | CSA | 1,57 | 10.86 | 0.002 | **17.32% larger** |
|  | *Roeboides* | Gatun 1935 vs. Mandinga 1911 | EA | 1,43 | 8.18 | 0.007 | 9.66% smaller |
|  |  | Gatun 1935 vs. Frijoles 1911 | EA | 1,45 | 4.70 | 0.036 | 4.29% larger |
|  |  |  | CSA | 1,40 | 7.10 | 0.011 | **14.5% larger** |
|  |  | Gatun 1935 vs. Chagres 1911 | CPD | 1,49 | 24.73 | <0.001 | 6.47% larger |
|  |  |  | EA | 1,51 | 22.25 | <0.001 | **12.43% larger** |
| **(ii) Invasion through time** | *Astyanax* | Gatun 1935 vs. 2013 | AD | 1,58 | 4.36 | 0.041 | 7.30% smaller |
|  |  |  | CPA | 1,57 | 17.44 | <0.001 | **24.7% smaller** |
|  |  |  | CSA | 1,57 | 4.58 | 0.037 | **28.6% smaller** |
|  |  | Chagres 1911 vs. 2013 | BD | 1,58 | 24.37 | <0.001 | 7.14% smaller |
|  |  |  | AD | 1,58 | 37.46 | <0.001 | 6.41% smaller |
|  | *Roeboides* | Gatun 1935 vs. 2013 | BD | 1,66 | 18.25 | <0.001 | 4.63% larger |
|  |  |  | BD_A_ | 1,66 | 14.50 | <0.001 | 4.07% larger |
|  |  |  | EA | 1,66 | 5.70 | 0.020 | 5.10% smaller |
|  |  |  | CSA | 1,56 | 52.79 | <0.001 | **27.7% bigger** |
|  |  | Chagres 1911 vs. 2013 | BD | 1,32 | 5.95 | 0.021 | 3.39% smaller |
|  |  |  | BD_A_ | 1,32 | 11.34 | 0.002 | 4.02% smaller |
|  |  |  | AD | 1,32 | 8.44 | 0.007 | 5.03% smaller |
|  |  |  | EA | 1,32 | 8.42 | 0.007 | **10.5% larger** |
| **(iii) Invasion across space** | *Astyanax* | Gatun 2013 vs. Bayano 2013 | BD | 1,48 | 5.045 | 0.0295 | 1.53% smaller |
|  | *Roeboides* | Gatun 2013 vs. Bayano 2013 | BD | 1,69 | 23.13 | <0.001 | 4.55% smaller |
|  |  |  | BD_A_ | 1,68 | 24.948 | <0.001 | 4.04% smaller |
|  |  |  | AD | 1,69 | 12.197 | <0.001 | 4.81% smaller |
|  |  |  | CPD | 1,69 | 15.915 | <0.001 | 5.58% smaller |
| **(iv) Temporal controls** | *Astyanax* | Trinidad 1911 vs. 2014 | BD | 1,60 | 80.96 | <0.001 | 8.37% smaller |
|  |  |  | CPA | 1,60 | 5.06 | 0.028 | 6.25% smaller |
|  |  |  | CSA | 1,60 | 16.09 | <0.001 | **13.6% larger** |
|  | *Roeboides* | Mandinga 1911 vs. 1992 | CPA | 1,27 | 6.17 | 0.020 | 0.28% larger |
|  |  |  | CSA | 1,27 | 6.27 | 0.019 | **13.9% larger** |

**Table S5.** Results of planned contrasts examining changes in linear traits of *A. ruberrimus* and *Roeboides* spp.

| **Taxa** | **Trait** | **df** | **T** | **p** |
| --- | --- | --- | --- | --- |
| 1. **Impoundment effect** | | | | |
| *Astyanax* | **BD** | **246** | **2.576** | **0.042** |
|  | AD | 246 | 1.906 | 0.212 |
|  | CPD | 244 | -1.642 | 0.349 |
|  | CPA | 244 | -1.884 | 0.222 |
|  | **EA** | **246** | **3.967** | **<0.001** |
|  | **CSA** | **244** | **-4.910** | **<0.001** |
| *Roeboides* | BD | 175 | -1.352 | 0.544 |
|  | BD_A_ | 173 | -1.701 | 0.316 |
|  | AD | 176 | 1.268 | 0.603 |
|  | CPD | 173 | -2.149 | 0.126 |
|  | CPA | 173 | -0.062 | 1.000 |
|  | EA | 175 | -0.804 | 0.889 |
|  | CSA | 162 | -0.821 | 0.881 |
| 1. **Invasion through time** | | | | |
| *Astyanax* | **BD** | **246** | **4.811** | **<0.001** |
|  | **AD** | **246** | **6.492** | **<0.001** |
|  | CPD | 244 | 0.687 | 0.934 |
|  | **CPA** | **244** | **2.612** | **0.038** |
|  | EA | 246 | -1.149 | 0.686 |
|  | CSA | 244 | 1.924 | 0.204 |
| *Roeboides* | BD | 175 | 0.078 | 1.000 |
|  | BD_A_ | 173 | 0.848 | 0.868 |
|  | AD | 176 | 1.558 | 0.403 |
|  | CPD | 173 | -1.727 | 0.302 |
|  | CPA | 173 | 0.177 | 1.000 |
|  | EA | 175 | -1.141 | 0.693 |
|  | **CSA** | **162** | **2.593** | **0.041** |
| 1. **Invasion across space** | | | | |
| *Astyanax* | BD | 246 | -0.221 | 0.999 |
|  | AD | 246 | 0.568 | 0.966 |
|  | CPD | 244 | 1.524 | 0.424 |
|  | CPA | 244 | 1.007 | 0.780 |
|  | EA | 246 | -1.510 | 0.433 |
|  | CSA | 244 | 2.874 | 0.018 |
| *Roeboides* | **BD** | **175** | **-5.523** | **<0.001** |
|  | **BD_A_** | **173** | **-5.258** | **<0.001** |
|  | **AD** | **176** | **-4.906** | **<0.001** |
|  | **CPD** | **173** | **-5.422** | **<0.001** |
|  | CPA | 173 | -1.108 | 0.715 |
|  | EA | 175 | -2.280 | 0.092 |
|  | CSA | 162 | -2.275 | 0.093 |
| 1. **Temporal Controls** | | | | |
| *Astyanax* | **BD** | **246** | **7.992** | **<0.001** |
|  | **AD** | **246** | **10.447** | **<0.001** |
|  | CPD | 244 | 1.245 | 0.619 |
|  | CPA | 244 | 2.077 | 0.146 |
|  | EA | 246 | 1.514 | 0.430 |
|  | **CSA** | **244** | **-4.422** | **<0.001** |
| *Roeboides* | BD | 175 | -0.270 | 0.998 |
|  | BD_A_ | 173 | 0.289 | 0.997 |
|  | AD | 176 | 1.375 | 0.527 |
|  | CPD | 173 | 0.828 | 0.878 |
|  | CPA | 173 | 2.073 | 0.149 |
|  | **EA** | **175** | **2.606** | **0.039** |
|  | CSA | 162 | -0.228 | 0.999 |

**Supplementary Figure Legends**

**Figure S1.** Morphological variation for A. ruberrimus through time. Data shown are means (± 2 SE) of size-adjusted residuals of PC1 - PC3 scores for body shape (refer to Table S2 for PC Loadings). Populations are coded by habitat (squares/diamonds for lakes, circles for large rivers and triangles for small streams), by perturbation type (white for pristine, light grey for impounded, dark grey for invaded and black for impounded + invaded), and by site classification (dotted line for control and solid line for impact). Lines were drawn between end-points to facilitate the visualization of temporal trends, but should be interpreted with caution, given that traits were not sampled continuously through time, as so the actual shape of the trend is unknown. A visual representation of the principal component axes (PC1 – PC3) for the geometric morphometric results are shown to the right of the corresponding time series. Each PC residuals axis is plotted in reference to the mean shape, distorting the grid where they differ. The distortion has been magnified by a factor of 3.

**Figure S*2*.** Morphological variation for *Roeboides* spp. through time. Data shown are means (± 2 SE) of size-adjusted residuals of PC1 - PC3 scores for body shape (refer to Table S2 for PC Loadings). Populations are coded by habitat (squares/diamonds for lakes, circles for large rivers and triangles for small streams), by perturbation type (white for pristine, light grey for impounded, dark grey for invaded and black for impounded + invaded), and by site classification (dotted line for control and solid line for impact). Lines were drawn between end-points to facilitate the visualization of temporal trends, but should be interpreted with caution, given that traits were not sampled continuously through time, as so the actual shape of the trend is unknown. A visual representation of the principal component axes (PC1 – PC3) for the geometric morphometric results are shown to the right of the corresponding time series. Each PC residuals axis is plotted in reference to the mean shape, distorting the grid where they differ. The distortion has been magnified by a factor of 3.

**Figure S3.** Time-series plots for A. ruberrimus showing the means (± 2 SE) of each population for each measured trait. Populations are coded by habitat (squares/diamonds for lakes, circles for large rivers and triangles for small streams), by perturbation type (white for pristine, light grey for impounded, dark grey for invaded and black for impounded + invaded), and by site classification (dotted line for control and solid line for impact). Lines were drawn between end-points to facilitate the visualization of temporal trends, but should be interpreted with caution, given that traits were not sampled continuously through time, as so the actual shape of the trend is unknown.

**Figure S4.** Time-series plots for Roeboides spp. showing the means (± 2 SE) of each population for each measured trait. Populations are coded by habitat (squares/diamonds for lakes, circles for large rivers and triangles for small streams), by perturbation type (white for pristine, light grey for impounded, dark grey for invaded and black for impounded + invaded), and by site classification (dotted line for control and solid line for impact). Lines were drawn between end-points to facilitate the visualization of temporal trends, but should be interpreted with caution, given that traits were not sampled continuously through time, as so the actual shape of the trend is unknown.

**Figure S5.** Linear Discriminant Analysis (LDA) for *Astyanax ruberrimus* linear traits.

**Figure S6.** Linear Discriminant Analysis (LDA) for *Roeboides* spp. linear traits.
